# Water affordability and human right to water implications in California

**DOI:** 10.1371/journal.pone.0245237

**Published:** 2021-01-20

**Authors:** Jessica J. Goddard, Isha Ray, Carolina Balazs

**Affiliations:** 1 Energy & Resources Group, University of California, Berkeley, California, United States of America; 2 Office of Environmental Health Hazard Assessment, California Environmental Protection Agency, Oakland, California, United States of America; Ministry of Health and Sports, MYANMAR

## Abstract

Water affordability is central to water access but remains a challenge to measure. California enshrined the human right to safe and affordable water in 2012 but the question remains: how should water affordability be measured across the state? This paper contributes to this question in three steps. First, we identify key dimensions of water affordability measures (including scale, volume of water needed to meet ‘basic’ needs, and affordability criteria) and a cross-cutting theme (social equity). Second, using these dimensions, we develop three affordability ratios measured at the water system scale for households with median, poverty level, and deep poverty (i.e., half the poverty level) incomes and estimate the corresponding percentage of households at these income levels. Using multiple measures conveys a fuller picture of affordability given the known limitations of specific affordability measures. Third, we analyze our results disaggregated by a key characteristic of water system vulnerability–water system size. We find that water is relatively affordable for median income households. However, we identify high unaffordability for households in poverty in a large fraction of water systems. We identify several scenarios with different policy implications for the human right to water, such as very small systems with high water bills and low-income households within large water systems. We also characterize how data gaps complicate theoretical ideals and present barriers in human right to water monitoring efforts. This paper presents a systematic approach to measuring affordability and represents the first statewide assessment of water affordability within California’s community water systems.

## 1 Introduction

Water affordability is central to water access but remains a challenge to measure. The United Nations General Comment No. 15 (GC15) on the human right to water defines water as economically accessible if the direct and indirect costs associated with water and sanitation do not impact a person’s access to other essential rights (e.g., food or shelter) [1, 2]. Following GC15, water affordability became established as a conceptual pillar of human right to water frameworks [1, 3–5]. The United Nations’ Sustainable Development Goal (SDG) report followed suit with the target to “achieve universal and equitable access to safe and affordable drinking water for all” by 2030 [6]. The SDG indicators do not include measures of water affordability, however. Thus there is a gap between the aspirations for affordable water for all and the availability of measures to track this aim. How can states leverage existing data to define and monitor water affordability at scales relevant to policy-making? Answering this question is necessary to support efforts to meet the SDGs and the human right to water across the globe. In this paper, we contribute to the emerging discussion on the meaning of drinking water affordability and the tractability of its measurement within a human right to water framework. We demonstrate our approach in California community water systems, where opportunities for state-level assistance to alleviate water affordability are actively being developed [7], but data constraints are persistent.

International work dominates safe water policy and research agendas [8, 9], but access to clean and affordable drinking water is a growing challenge in the United States [10, 11]. The severity of water access inequities in the U.S. jarred national consciousness with the uncovering of Flint's lead crisis [12] and of thousands of water shut-offs in Baltimore and Detroit due to unpaid bills [13]. In the U.S. overall, water prices are rising faster than inflation in urban areas [14]. Additionally, water affordability is entwined with inequitable contaminant exposure. Unaffordable water bills compound and perpetuate water quality problems, leading to a “joint burden” on households [15]. Disparities in drinking water access persist in part because of inequities in infrastructure [16], high tap water and bottled water costs paired with low ability-to-pay [17, 18], low technical, managerial and financial capacity [15], and rate-design that inadequately addresses households’ ability-to-pay [19].

How to measure affordability is an active debate in the US [20–22]. The dominant approach is to measure the cost of drinking water relative to income. This ratio, compared against a specified threshold, identifies water (un)affordability [8, 9, 23–28]. Several studies highlight water affordability challenges in California [14, 22, 29, 30], Michigan [31], the US-Mexico border in Texas [24], and the U.S. overall [25–27]. Most of these studies tell a similar story: lower-income households or communities, even in relatively rich contexts, frequently face unaffordable water costs.

Two questions frame this paper: How can we measure water affordability in California’s CWS to meet (and monitor) the human right to water? What do these measures tell us about water affordability in and across differently-sized CWSs? To balance analytical rigor with usability, we co-produced our research design and resulting measures with California Environmental Protection Agency’s (EPA) Office of Environmental Health Hazard Assessment, which is developing approaches to evaluate the human right in California using publicly available data in concert with broader state efforts on low income rate assistance [7, 32, 33]. Our questions required theoretical, methodological and empirical work that we undertook in two stages.

The first stage was *affordability measure development*, leveraging publicly-available, routinely-collected data. We identify key dimensions relevant to water affordability that are grounded in theoretical and practical considerations (Section 2) and use these dimensions as guidelines to develop a set of affordability measures and household poverty indices (Section 3). Concerns over how affordability measures should incorporate economically vulnerable groups are paramount, but additional debates include questions of spatial and temporal scale, the volume of water use that should be measured, and which criteria should be used to judge affordability. Assessing these dimensions in measure development allows us to situate our evaluation of affordability ratios within existing policy efforts, research frontiers and critiques of what affordability ‘should’ measure. Here we focus on California’s ~2,900 community water systems (CWS). CWSs are public water systems that serve water year-round to at least 25 people or have 15 or more service connections (Health & Safety Code 2017). We focus our work on the cost of domestic water used for drinking, hygiene, and cooking in California’s CWSs, and do not explicitly incorporate wastewater and sanitation costs (Many water systems–especially larger utilities–provide a combined water, sewer and stormwater bill to households. Sewer and stormwater costs may exceed the cost of drinking water supply itself. Our affordability estimates use data from the state’s electronic Annual Report (eAR) that explicitly asked for the cost of 6HCF of water as part of a survey on water rates for the state’s Drinking Water Program.), as this data is not readily available for systems. We use system-level metrics to capture household water bill burdens at specific income levels, as this is the most granular level of data publicly available and is also consistent with broader human right to water efforts in the state [32].

In the second stage, we used these measures to conduct a *statistical analysis* of affordability estimates by water system size (Section 4). In California and beyond, funding for safe and affordable water is frequently determined by CWS size as a common delineator of a water system’s technical, financial, and managerial capacity [34]. Disaggregating affordability results by system size can thus yield insight into potentially disparities relevant to the human right to water. We find gaps in data availability, especially for smaller systems, so we evaluate bias and characterize systems with missing affordability data in an effort to support California’s efforts to monitor the human right to water for all people. We also address data reliability through sensitivity analyses of water bill and income data. These efforts facilitate the inclusion of smaller systems, where population counts are small but the challenges for safe water access are big [35].

We select California as a case study for several reasons. Water bills across the state increased by 42 to 47 percent over the previous two decades, disproportionately impacting households served by smaller systems [36]. In 2012, California was the first U.S. state to establish safe, clean, affordable, and accessible water as a human right (Assembly Bill 685) [37]. State efforts to support water systems in alleviating quality or affordability challenges have focused on funding support for small socioeconomically disadvantaged systems [34]. An estimated 20% of households eligible for water subsidies actually receive assistance, but the State Water Resources Control is investigating policy options for state-wide household-level bill assistance [7, 33]. Grassroots organizations, water suppliers, and communities are actively debating the meaning of water affordability and programs through public comment processes. However, the human right to water bill does not outline a preferred process to assess and track the human right to water. This paper contributes to this discussion by offering multiple measures of water affordability to support monitoring efforts towards the realization of the human right to water in California.

## 2 Measuring water affordability: Approaches and key dimensions

In this section we discuss approaches to water affordability measurement (Section 2.1) and then discuss a set of key dimensions of water affordability distilled from an extensive review of gray and academic literatures (Section 2.2). Affordability measures for human right to water monitoring are primarily ratio-based measures, which calculate water costs as a proportion of incomes [5, 9]. Key dimensions that influence measurement include: spatial and temporal scale, water to meet basic needs, counting all the costs, available income, criteria for affordability, and social equity. Using these dimensions to guide our own development of affordability ratios (Section 3), we show how theoretical ideals are often at odds with practical data limitations. Indicating where these disjunctions occur in our own affordability ratios makes clear the data gaps in human right to water monitoring efforts. These dimensions are a methodological contribution to the development of transparent affordability ratios where data is incomplete.

### 2.1 Approaches to water affordability measurement

The dominant affordability ratios are: *conventional affordability ratios* (CAR) and *potential affordability ratios* (PAR) [38]. The CAR measures average water costs relative to income and indicates unaffordability when this ratio exceeds a specified threshold. The PAR measures water costs for a specified volume for meeting basic needs and indicates unaffordability when ‘basic needs’ water costs exceed a given proportion of income. Ideally studies should include all costs of accessing water and sanitation in a human right to water context, but these are often separated due to data limitations [1, 2, 39].

The PAR offers the benefit of 1) assessing affordability for water to meet basic as opposed to discretionary needs and 2) allowing for a comparison of the burden of water bills for a fixed volume across multiple households or water systems [23, 38, 40]. An extension of the PAR, AR_20_, is the affordability ratio at the 20^th^ income percentile [25]. AR_20_ evaluates water and (if available) wastewater bills for the lowest income quintile, less essential expenditures such as food and rent. Developed for metropolitan regions, AR_20_ assumes that the 20^th^ percentile reflects poverty-level incomes; however, this may not hold in water systems where the vast majority of households are well-off. Measuring AR_20_ requires analysts to define and quantify what constitutes essential expenditures beyond water. Beyond ratio measures, affordability has been measured by evaluating the difference in income after paying for utilities in comparison with a poverty level income (the residual income approach) [38, 41].

### 2.2 Key dimensions of affordability ratio measurement

Below, we summarize key dimensions of affordability ratio measurement, discuss the critiques associated with each, and identify approaches to address their limitations. These dimensions are distilled from a broader literature review on water affordability measures from academic and policy literatures [42].

#### 2.2.1 Spatial and temporal scale

Water affordability is experienced at the household scale [38, 43–45], but policy measures typically use aggregated data at scales from water systems [46] to nation states [47]. Most researchers advocate disaggregating results by income groups to better capture variation in affordability within larger geographies. Evaluating water affordability over time or evaluating future rates [27, 48] is less common, but can illuminate temporal dynamics that cross-sectional studies do not capture.

#### 2.2.2 Water to meet basic needs

Studies based on average household water use risk under- or over-estimating affordability problems because they do not focus on essential water use [38]. Human right to water efforts are concerned with filling basic needs, as opposed to luxury water uses (e.g., filling pools). This dimension makes it necessary to decide what constitutes a minimum volume. Several approaches exist, including: 1) determining basic needs water from demand functions [28, 49]; 2) estimating minimum water requirements for universal norm-setting [50, 51]; and 3) deriving location-specific estimates of water use based on ‘reference budgets’–or the minimum volumes needed for washing, cooking, hygiene, and consumption [4, 45].

#### 2.2.3. Counting all the costs

Affordability ratios ideally include the full set of water-related costs that households incur. This includes water for cooking, drinking, hygiene, sewer, stormwater, and any additional costs that households incur. Examples of additional costs include fees incurred during drought [52] or coping costs (such as bottled water) on top of the water bill when the main water source is not safe [18].

#### 2.2.4. Available income

The denominator of an affordability ratio defines the amount of money a household has available to spend on water. In the absence of household-level income and expenditure data, studies use proxy measures such as gross income. More precise measures of available income include disposable income [8, 23, 38], or disposable income less (modeled) estimates of essential expenditures, which results in an estimate of discretionary income [25]. Discretionary income needs to be accounted for by income level, otherwise the affordability situation of a middle- or high-income households with high expenditures may be conflated with that of lower-income households. In low-income household surveys, expenditures may be a more accurate measure of available income than reported income due to the nature of lower-income jobs [53].

#### 2.2.5. Criteria for affordability

Criteria to evaluate affordability ratios range from water bills being 1.5% to 10% of income, varying by whether they include both drinking water and sewer [9, 34, 54, 55]. Affordability criteria have been developed at the household and community-level. EPA’s federal affordability frameworks for drinking water (1998) and wastewater (1997) discuss household affordability as an aspect of a water system’s financial capacity, driven by cost-recovery concerns [54, 56]. Affordability for the ‘median household’ is understood to reflect a proxy of a community’s capacity to bear water costs. These reports suggested affordability criteria for water and sewer bills as 2.5% and 2% of median income levels within water systems, respectively. In practice, states use lower thresholds in their assessments of system-level affordability than EPA’s standard. In California, the State Water Resources Control Board has used a threshold of 1.5% of median household income to provide financial aid to lower-income water systems. For households, the most frequently cited threshold for water and wastewater bills as a percentage of disposable income is 3% [57, 58]. Recently, 10% was proposed to evaluate drinking water and sewer costs relative to discretionary income for the 20^th^ percentile income level within a water system [25]. Other researchers, our team included, present affordability estimates disaggregated by multiple categories (e.g., system size) and interpret affordability for specific income levels within a water system as a spectrum. without declaring a particular threshold as a bar [32, 59]. This approach does not preclude the use of thresholds but rather emphasizes looking at several affordability results, as different measures and thresholds have different policy implications in local contexts.

#### 2.2.6. Social equity

Affordability for the human right to water demands attention to economically vulnerable households and other marginalized communities. The U.S. EPA framework for water affordability has been critiqued for focusing only on median income, thereby underrepresenting economically vulnerable groups [60, 61] or ignoring income variation within larger geographies. Recent recommendations to EPA’s frameworks argue that affordability measures should focus on low-income households [20]. Options to address this critique include: 1) estimating affordability for lower-income households [25, 38, 58] and across income groups [43, 45, 59]; and 2) with caveats, using affordability ratios based on median income in very low-income communities.

## 3 Affordability measure development

This section addresses our first research question–How can we measure water affordability in California’s CWS to meet (and monitor) the human right to water? To answer this, we developed three PAR affordability ratios that collectively address several aspects of the key dimensions presented in Section 2. The ratios capture the impact of water bills on households (Section 3.1) earning three different income levels (median income and two poverty-level incomes) across California (Section 3.2–3.3) using data at the water system *scale* for monitoring California’s human right to water [32]. We also developed two poverty level indices to indicate the magnitude of poverty-level water affordability challenges across systems (Section 3.4). We discuss how these measures can be collectively used to evaluate affordability (Section 3.5) and then summarize how the measures relate to the key dimensions of affordability identified below (Section 3.6).

### 3.1 Water bills in community water systems

Our measures were co-produced with OEHHA, using available data and the literature to guide our measure development. We used 2015 water bill and income data as that was the most recently available during our study period. We obtained a list of 2,901 CWS that were active in 2015 and their geographic boundaries from the Public Health Institute’s Water Boundary Tool [62]. To capture the *cost of water* to households, we used reported water bills at fixed water volumes from the 2015 California State Water Resources Control Board’s Electronic Annual Report (eAR) dataset. Water systems determine water bills using different rate structures–which vary across the state from a flat fee to a variable usage rate–and any added fee or subsidy they may include. The eAR survey asks systems to report the average residential customer water bill per month in dollars for 6, 12, and 24 hundred cubic feet (HCF). The average water bill at these volumes should include, in theory, fixed and variable costs of domestic water to households. However, as it is a reported bill and not an explicit calculation conducted by the authors of this paper, we are limited in our ability to discern whether rates include all additional fees or subsidies. The survey is conducted for the state’s Drinking Water Program, and our own discussion with many systems led us to believe these rates are unlikely to include sewer or stormwater fees. We evaluate affordability by using water bills reported for 6 HCF, as this volume was the closest to approximating a minimum volume of *water to meet basic needs* and aligns with various agency efforts to measure affordability [32]. 6 HCF equals 4,488 gallons per month, or approximately 37 gallons per capita per day (gpcd) in a four-person family and 49 gpcd in a three-person family. This range (37–49 gpcd) aligns with California’s conservation goals of 55 gpcd (California Water Code §10608.2) and recommendations to evaluate water affordability at 43 gpcd in California’s human right to water [4]. We note that it is substantially higher than international estimates for basic needs, which are closer to 26 gpcd [51]. It is, of course, possible to argue in favor of other ‘basic needs’ volumes, or against affordability criteria being confined to basic needs water at all, but in the California context the 6 HCF floor is useful both because these data are available annually and because it covers the state’s human right recommendation (assuming a 4-person household). Reported water bill data were cleaned using R Version 3.5.1 [63] and adjusted, as needed, in accordance with predetermined data cleaning criteria (S1 File).

### 3.2 Affordability measure 1: Households earning median household income (AR_MHI_)

Our first affordability ratio evaluates water bills for 6 HCF for households earning at the median household income (MHI) of a water system’s income distribution (AR_MHI_). High values of AR_MHI_ can signal that water affordability is a problem for a majority of households within the system (Eq 1):

$${AR}_{MHI}=\frac{Monthly\;Water\;Bill\;at\;6\;Hundred\;Cubic\;Feet*12\;months/year}{Annual\;Median\;Household\;Income\;in\;Water\;System}\times 100$$ (1)

We estimated an MHI for each water system using block group MHI data from the American Community Survey (ACS) (5-year estimates; 2011–2015). Because CWSs do not share boundaries with census-designated geographies, we apportioned census block group data to water system boundaries using an aerial-household weighting method [64] as follows: We first estimated the proportion of census block area overlapping with CWS boundaries in ArcGIS. This aerial proportion was used to weight the number of households in blocks intersecting CWS boundaries. Weighted household counts were then summed to their respective block groups and water systems, resulting in an estimate of the number of households served by a water system within each block group and a total number of households by water system. These estimates were combined with MHI data by block group to calculate a weighted average MHI for each water system (see S3 File for details of the weighting method). Finally, we explored the reliability of this approach given census errors (Section 4.3).

### 3.3 Affordability measures 2 and 3: Households earning county poverty (AR_CP_) and deep poverty incomes (AR_DP_)

AR_MHI_ does not indicate water bill impacts on economically vulnerable households unless a majority of households in the water system are low-income. We therefore developed affordability ratios for two types of low-income households those earning at county poverty levels (Eq 2: AR_CP_), and those in ‘deep poverty’ for their counties (Eq 3: AR_DP_). These income levels are better proxies for *available income* in the denominator relative to median household income because they define a minimum disposable income required to stay out of poverty for the county.

$${AR}_{CP}=\frac{Monthly\;Water\;Bill\;at\;6\;HCF\;*12\;months/year}{County\;Poverty\;Level\;for\;Water\;System\;}\times 100$$ (2)

$${AR}_{DP}=\frac{Monthly\;Water\;Bill\;at\;6\;HCF\;*12\;months/year}{\frac{1}{2}(County\;Poverty\;Level\;for\;Water\;System)}\times 100$$ (3)

The county poverty level (CP) reflects essential household expenses, or a minimum disposable income, adequate for a household of four to stay out of poverty within their county. Deep poverty (DP) is defined as 50% of CP to capture extreme economic vulnerability. Deep poverty provides a snapshot of the most vulnerable households and is used as a benchmark for severe poverty by the Census and the Public Policy Institute of California (PPIC). Both measures adjust for key differences in expenses across counties, such as housing costs [65]. We acquired county poverty data from the PPIC and assigned every water system the poverty and deep poverty level of its respective county for 2015. Of California’s 58 counties, 38 have unique poverty levels. The PPIC divides the remaining 20 counties into three groups with equal poverty levels (due to census data suppression criteria).

These measures enable us to incorporate *social equity* by using multiple income levels within and across systems. However, our measures do not remove essential non-water expenditures from the denominator. Thus, even when AR_CP_ and AR_DP_ are low (i.e., water is relatively affordable), we cannot identify if households are compromising other needs.

### 3.4 Household poverty indices to complement affordability measures

While fifty percent of households face *at least* AR_MHI_ by consequence of using median income levels, the extent of households facing at least AR_CP_ or AR_DP_ is not known. To capture this, we estimated the number of households within a water system at or below the county poverty level (HH_CP_) and deep poverty level (HH_DP_). We divided these sums by the total number of households in each water system to calculate percentages, resulting in two household poverty indices (Eqs 4 and 5):

$${HH}_{CP}=\frac{\sum Households\;in\;Water\;Sytsem\leq County\;Poverty\;Level\;}{Total\;Households\;in\;Water\;System}\times 100$$ (4)

$${HH}_{DP}=\frac{\sum Households\;in\;Water\;Sytsem\leq County\;Deep\;Poverty\;Level}{Total\;Households\;in\;Water\;System}\times 100$$ (5)

To estimate HH_CP_ and HH_DP_, we used census block group household count estimates of: 1) total number of households and 2) the number of households within each of sixteen income levels. We applied the aerial-household weighting method described above to obtain system-level household count estimates. Given that the Census bins the number of households into discrete income levels (e.g., $15,000-$25,000), we used linear interpolation in R Version 3.5.1 to arrive at the number of households within each system falling at or below the county poverty and deep poverty levels [63].

### 3.5 Criteria for affordability

In practice, *criteria for affordability* are essential to assess affordability burdens and to allocate resources. No single threshold has been explicitly accepted as a measure of affordability in California (though numerous thresholds are used). Any state-level determination of affordability criteria will eventually be a matter of policy debate and stakeholder engagement. Therefore, we do not select a single threshold to evaluate the three affordability measures. We interpret affordability as being on a spectrum from more affordable (i.e., lower AR values) to less affordable. We also use the two household poverty indices to contextualize the poverty-level affordability measures. We compare our results with existing thresholds (e.g., 1.5% of MHI, and 3% of disposable income) for illustrative purposes, but focus instead on the distribution of our results within and across CWS. In the Supplemental Information we show how the number of systems with unaffordable water changes under different ratios (S8 Table in S8 File).

### 3.6 Affordability measures in relation to key dimensions

Table 1 indicates how, together, the three affordability measures developed relate to the key dimensions of water affordability, reflecting essential needs water, economically vulnerable groups, and indicate potential inequities across and within systems. Because these measures do not include sewer or sanitation costs, they only partially address the human right to water. The ratios developed here are specific for California, but the approach to developing measures in conversation with key dimensions of affordability are useful for other policy contexts.

**Table 1:** Summary of key dimensions for the development of affordability ratios.

Dimension	Median Affordability Ratio (AR_MHI_)	Poverty Affordability Ratio (AR_CP_)	Deep Poverty Affordability Ratio (AR_DP_)
Spatial and Temporal Scale and Scope	California is the extent of the study and water systems are the unit of analysis, which aligns with current human right to water efforts in California and is the scale with the most comprehensive, state-wide data.		
	*Affordability is approximated for three household-level incomes*, *but ratios are not evaluated for each household*, *which is likely impractical for state-wide monitoring*.		
	*Affordability analysis is not temporal. However, metrics are part of a human right to water monitoring effort and will be measured over time [32].*		
Water for basic needs	Water bill evaluated at 6 HCF per month to approximate the volume of water needed to meet basic needs for households and to parallel California conservation goals.		
	*Variations in basic needs for vulnerable groups (e*.*g*. *families with babies or medical needs)*, *larger or smaller household sizes*, *and/or differing geographies are not addressed*.		
Costs	Use of reported average water bills for 6 HCF per month, which includes the price of water and any fees or subsidies included by the water system.		
	*Sewer costs and stormwater costs are not included due to incomplete statewide data at water system scale*. *If households obtain basic needs water from alternate sources (e*.*g*. *bottled water)*, *these are not reflected in water bills*.		
Available income	Median household incomes do not capture the heterogeneity of incomes within water system.	County poverty levels incorporate cost of living and minimum essential needs budget, approximating disposable income at poverty levels.	Deep poverty levels reflect households with an extreme income constraint, at *half* disposable income for poverty level households.
	*Median household incomes are gross income levels and do not fully reflect available income because they include taxes and other expenditures*.		
		*County poverty level ARs do not evaluate how water costs impact other essential expenditures*.	*Deep poverty level ARs do not evaluate how water costs impact other essential expenditures*.
Criteria for affordability	Study assesses distribution of data and disaggregates analysis by system size. Measures are part of a broader human right to water effort that will analyze trends over time in California.		
	A single affordability threshold is not selected.		
Social equity	Income at 50^th^ percentile of low-income system can indicate concentrations of low-income households.	Social equity is partially addressed through a focus on economic vulnerability by focusing on households earning at the county poverty level, an income level that approximates disposable income for vulnerable households.	Social equity is partially addressed through a focus on economic vulnerability, which is explicitly addressed by focusing on households at the deep poverty level, an income level that approximates disposable income for extremely vulnerable households.
	*Social equity is not explicitly addressed*.		
	Taken together, all three ratios and the poverty indices reflect the income distribution below the median income level within each system. Social equity is addressed in a comparison of water bill burdens across income levels.		
	*Disaggregation by known social-demographic factors in water access*, *like race/ethnicity*, *are not addressed*.		

Plain text summarizes how the ratio used in this paper addresses the dimension. Text in italics indicates aspects of affordability *not* captured by these measures (i.e., their limitations).

## 4 Statistical analysis of affordability measures

This section uses the measures developed in Section 3 to answer our second question: What do these measures tell us about water affordability in and across differently-sized CWSs? We estimated these measures for CWS with data in California and investigated the association of system size with each system’s affordability ratios (AR_MHI_, AR_CP_, AR_DP_) using generalized linear regression models. We also evaluated household poverty indices (HH_CP_ and HH_DP_) across CWS by system size using an analysis of variance. Systems are considered very small if they serve 25–500 people, small if 501–3,300 people, medium if 3,301–10,000, large if 10,001–100,000, and very large if >100,000 people [following 66].

Because our study encountered high levels of missing data, we evaluated bias and potential confounders of missing data (Section 4.1) prior to implementing our statistical assessment of affordability estimates by system size (Section 4.2). To conclude, we address data reliability concerns by conducting a sensitivity analysis (Section 4.3). All analyses were conducted in R Version 3.5.1.

### 4.1 Addressing missing water bill and income data

1,400 of 2,901 CWS–representing about 5% of California’s community water system population–were missing relevant data to estimate affordability. As missing data can lead to biased conclusions, we evaluated sample bias and attempted to reduce measured confounding of missingness on affordability outcomes in our statistical models of affordability by system size. Across a variety of social-demographic and water system characteristics, we found the primary bias due to missing data was an 18% under-representation of water systems that are very small or small relative to the overall population (S5 File). Methods to address missingness include multiple imputation, inverse probability weighting, and complete case, appealing to the controlling for confounding caused by missingness by adjusting for appropriate variables assuming missing at random [67]. As our study explores how system size impacts affordability but we were missing data for very small or small systems, we quantified potential confounding by using proxies for affordability to predict missingness *within* system size categories. This approach relies on the assumption that systems were missing data at random *within* size categories. Thus, we first stratified the community water system list by four system size categories. We collapsed the very large and large system size category (10,000+ people) to balance group size in our statistical analysis. We then modeled missingness (i.e. if a system was missing water bill or income data) within each size category using variables the literature suggests might be correlated with affordability. Where these proxies indicated a marginal effect on missingness within size categories (odds ratio greater than 1), we included them in a generalized model of affordability to reduce variance on account of measured confounding of missingness *and* affordability.

To model missingness we coded systems with a 1 if they were missing water bill or income data and a 0 if they had data. We ran seven separate logistic regressions within each of the four size categories (S5 File). The independent variables included water source type (surface water or groundwater), water system ownership (private or public), and water board governance region as designated in the Safe Drinking Water Information System (SDWIS). Other potential confounders included percent renters, percent households under twice the poverty level, and percent people of color (all categories combined excluding white) from the ACS (5-year averages, 2012–2016). ACS variables were aerially apportioned to water systems following the same method described for median household income but using population instead of household weighting. When any one potential confounder was found to significantly increase the odds of a system having missing data at alpha = 0.05, we incorporated the variable as a covariate in the analysis of affordability ratios by system size. In theory, this approach will reduce confounding of missingness on affordability when analyzing the affordability estimates.

### 4.2 Statistical model for affordability and household poverty indices estimates

Using the variables identified as confounders of missingness above, we modeled each affordability ratio in a generalized linear model first including, and then excluding, the system size variable. Low Akaike information criterion (AIC) and a significant F test (alpha = 0.05) comparing the two models served as an omnibus test for the influence of size on affordability ratios. We then estimated adjusted mean affordability ratios and 95% confidence intervals by system size (using the *ggeffects* package in R) and conducted Tukey’s post-hoc tests adjusting for multiple hypothesis testing using the *multcomp* package [68].

Using our estimated household poverty indices (HH_CP_ and HH_DP_), we performed One-way ANOVA regressing each poverty index against water system size to understand the extent to which poverty-level households were more or less concentrated by water system size. This was done for the entire system list with geographic boundaries (n = 2,882) and the sample list (n = 1,501). We used Welch’s test to account for unbalanced groups and unequal variances among groups with the *userfriendlyscience* package in R Version 3.5.1 [69]. Where Welch’s ANOVA was significant, we conducted Games-Howell post-hoc tests to evaluate difference of means for household poverty indices across system sizes.

We assessed normality and variance of all results–AR_MHI_, AR_CP_, AR_DP_, HH_CP_ and HH_DP_–for the full distribution and by system size. We log transformed all affordability ratios and square-root transformed HH_CP_ and HH_DP_ to account for non-normality. Residuals from models were evaluated for normality using Shapiro-Wilks tests. Shapiro-Wilks tests are highly sensitive to deviations from normality so QQPlots were consulted given the large sample size [70].

### 4.3 Addressing data reliability through sensitivity analysis

Given the absence of any prior state-wide assessment of eAR accuracy, missing data, and reliability concerns with Census estimates, we explored the sensitivity of our model findings to unreliable data. We investigated potentially unreliable water bill and/or census income data and then ‘flagged’ systems as for removal in a sensitivity analysis if they had unreliable data. For water bills, we used an adjusted box plot for skewed distributions to identify potential outliers [71]; this approach yielded upper and lower limits on water bills based on their distribution. Water systems whose water bills were below or above the fence were contacted by phone to verify the accuracy of the data (S2 File).

Systems were flagged as having unreliable Census data in two ways. First, CWSs were flagged when they had Census income data missing for more than 15% of households with block group data (S4 File). Second, we assessed census data reliability for systems falling within one block. Systems were flagged if the census MHI estimate: 1) had a coefficient of variation greater than 50 *and* 2) the standard error of the estimate was greater than the mean standard error of all California block groups for the estimate (OEHHA, 2017). We flagged unreliable data in the HH_CP_, and HH_DP_ estimates if 20% of the underlying household count estimates across income levels were unreliable by these criteria (S4 File). We ran all models and analyses with all systems that had data, and then excluded systems with potentially unreliable data. This allowed us to evaluate the sensitivity of our results to data quality concerns.

## 5 Results

In this section, we present the results of our statistical analysis. We summarize the final study sample list (Section 5.1) and provide descriptive statistics for our calculated affordability and poverty indices estimates across all systems (Section 5.2). Sections 5.3 and 5.4 present our affordability ratio and household poverty index estimates, respectively, disaggregated by size.

### 5.1 Final study list of community water systems

A total of 2,901 California CWSs were active in 2015. Of these, 2,882 systems have water system boundary data; the 19 systems missing boundary data are primarily prisons that did not charge or report water bills. Of the 2,882 remaining systems, 1,369 systems had missing water bill data and 31 systems had missing income data. This resulted in 1,501 systems with water bill data at 6 HCF and income data to assess affordability ratios and household poverty indices (S1 Fig in S1 File). These systems serve approximately 33.2 million Californians, or 95% of the state’s population served by CWSs in 2015.

Our assessment of potential bias due to missing data indicates that the sample is relatively representative of the full population of water systems across key system and social-demographic characteristics, but that it underrepresents very small systems by 18.5% and CWSs with very low MHI levels by 5% (S5 File).

Our assessment of data reliability by excluding water bill outliers or unreliable income data did not affect the overall trend of results presented below. We identified 148 systems with unreliable data due to the water bill outlier assessment (n = 98), income data missing for more than 15% of households (n = 46), or census reliability exclusion criteria for MHI estimates (n = 8). Four systems fell into more than one category. An additional 227 systems were flagged as having potentially unreliable household count estimates for the poverty indices. There were some differences in post-hoc test results for affordability ratios and household poverty indices, which we discuss below (with further details in S7 File).

### 5.2 Descriptive statistics: Water bills, income levels, and affordability results

The variables for constructing the three affordability ratios and two household poverty indices are water bills, median household income, the county poverty income level, and the county deep poverty income level (Table 2). For 2015, monthly water bills for 6 HCF spanned three orders of magnitude in our sample, ranging from $3.06 to $466.00. The average reported bill across systems was $52.44 (median = $41.42). Median household incomes across the state ranged from $17,400 to $250,000. The range of county poverty levels–i.e., the minimum income needed to remain out of poverty for a family of four–and deep poverty levels were $23,700 to $36,200 and $11,900 to $18,000, respectively. The distribution of poverty incomes was relatively similar between our sample and the overall CWS list. In total, there were 41 systems (2.7% of 1,501) in the sample with MHI below their respective county’s poverty level, compared with 141 systems (5.9% of 2,882) in the full study list.

**Table 2:** Summary statistics for water bills and income data in affordability study sample and full community water system list for 2015.

	Study Sample (n = 1,501)	All CWSs (n = 2,901)
	**Monthly Water Bill– 6 HCF ($)**	
	n = 1,501	
*Average ± SD*	52.44 ± 41.35	Full distribution not known
*Median (IQR)*	41.42 (29.19, 61.33)	
*Minimum*	3.06	
*Maximum*	466	
* *	**Median Household Income ($)**^**‡**^	
	n = 1,501	n = 2,813^a^
*Average ± SD*	64,600 ± 29,200	61,800 ± 27,900
*Median (IQR)*	58,300 (44,000, 78,400)	55,600 (41,400, 76,400)
*Minimum*	17,400	13,400
*Maximum*	250,000	250,000
* *	**County Poverty Level ($)**^**‡**^	
	n = 1,501	n = 2,882^b^
*Average ± SD*	28,200 ± 3,300	27,800 ± 3,300
*Median (IQR)*	27,900 (25,100 30,500)	27,000 (25,000 30,300)
*Minimum*	23,700	23,700
*Maximum*	36,200	36,200
* *	**County Deep Poverty Level ($)**^**‡**^	
	n = 1,501	n = 2,882^b^
*Average ± SD*	14,100 ± 1,600	13,900 ± 1,600
*Median*	14,000 (12,500, 15,200)	13,500 (12,500, 15,200)
*Minimum*	11,900	11,900
*Maximum*	18,100	18,100

^**‡**^All income data is rounded to the nearest $100 in 2015 dollars.

^**a**^2,813 systems with available data; 69 water systems had no median household income data available; 19 systems had no spatial boundaries to intersect with census data.

^**b**^2,882 systems with available data; 19 systems with no spatial boundaries to intersect with census data.

Descriptive statistics of affordability ratios estimated for the three income levels are summarized in Table 3. Across systems, AR_MHI_ ranged from 0.04% to 13.2%. A majority of systems had a relatively low AR_MHI_–the 75^th^ percentile AR_MHI_ was 1.3%. Of the 281 systems with AR_MHI_ greater than California’s recommended threshold of 1.5%, 172 systems (or 11% of 1,501) had an MHI considered disadvantaged (less than 80% of the statewide MHI). For the 41 water systems with MHI *lower* than the respective county poverty level, the average affordability ratio was notably higher, at 3.1%.

**Table 3:** Summary statistics for affordability ratios and household poverty indices estimated at the community water system scale (n = 1,501) for 2015^‡^.

	Affordability Ratio for households–Median Household Income	Affordability Ratio for households–County Poverty Level	Affordability Ratio for households–Deep Poverty Level	% Households in water system at or below County poverty level*	% Households in water system at or below Deep poverty level*
	AR_MHI_ (%)	AR_CP_ (%)	AR_DP_ (%)	HH_CP_ (%)	HH_DP_ (%)
*Average (SD)*	1.1 ±1.0	2.2 ± 1.7	4.5 ± 3.5	24 ± 12	10 ± 7
*Median (IQR)*	0.9 (0.6, 1.3)	1.8 (1.3, 2.7)	3.6 (2.5, 5.3)	23 (15, 31)	9 (5, 13)
*90th percentile*	2.1	3.9	7.9	40	17
*99th percentile*	5.1	9.5	19	60	30

^‡^Estimates are rounded to the nearest tenth of a decimal.

19% of systems in the sample had AR_CP_ greater than the UN’s suggested threshold for water and sewer services of 3% of disposable income (S8 Table in S8 File). However, AR_CP_ does not include sewer costs and thus more systems likely exceed the 3% threshold. The average household at the county poverty level had twice the affordability ratio of the average household at the median income (average AR_CP_ = 2.2% and average AR_MHI_ = 1.1%). As the deep poverty level is by definition half the county poverty level, the AR_DP_ was 4.5% on average. A quarter of water systems (the 75^th^ percentile) had AR_CP_ and AR_DP_ greater than 2.6% and 5.3%, respectively.

Even average water bills can drive high affordability ratios in low-income households. For households in deep poverty paying at or below the sample mean of $52.44 per month, the average affordability ratio was 2.8%. Therefore the average drinking water bills for deep poverty households comprised nearly the entire 3% threshold for water and sanitation as a proportion of disposable income. Overall, the highest poverty-level affordability ratios were in systems with the highest water bills. Among the 148 systems with AR_CP_ greater than 4% (the top 10^th^ percentile of AR_CP_), the median water bill for 6 HCF was 2.4 times the state-wide average, or $125.10 per month. This suggests that while poverty incomes drive unaffordability in many cases, high water bills are also of concern.

### 5.3 Affordability ratios by water system size

**Fig 1:**
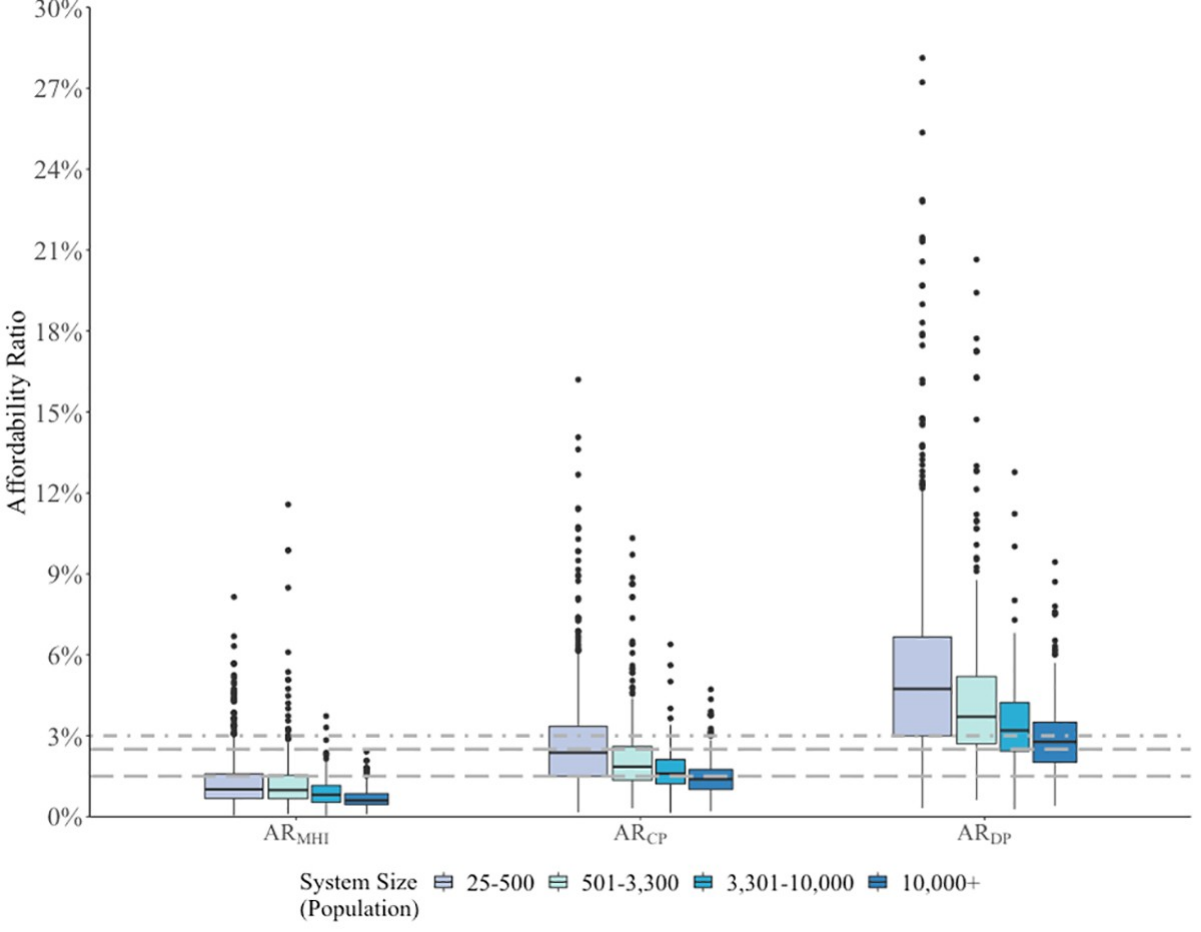
Box plots for crude (unadjusted) affordability ratios across income levels, by systems size. Affordability ratios (AR) estimate monthly water bills for 6 HCF relative to three income levels: AR_MHI_ = median household income level; AR_CP_ = county poverty level; and AR_DP_ = deep poverty level. Highest value for AR_DP_ (45.85%) not shown for readability. Long dashed lines represent common thresholds for households earning median household incomes within a water system–1.5% [34] and 2.5% [54]. Dot-dashed lines represent the commonly referenced 3% threshold–which compares water bills (sometimes including sanitation) to income (often disposable income) [57, 59].

Of the 1,501 systems in our study, 661 systems (44%) are very small, 304 (20%) are small, 166 (11%) are medium, and 370 (25%) are large. There is a clear gradient in affordability ratios based on water system size (Fig 1). Large systems had a tighter and lower distribution of water affordability ratios than very small and small systems across income levels. This may reflect diseconomies of scale in small systems.

In our models of missing systems by size, we identified water system characteristics (ownership, water source type) and region that were significantly associated with missingness within the very small size category (measured as odds ratios with p < 0.05) (Table B in S5 File). Social-demographic characteristics were also significantly associated with missingness, but the effect sizes were small (i.e. odds ratios of a system having missing data between 1 and 1.02 for an increase of 1 percent in social-demographic variables). We included all of the variables in linear models regressed against each log-transformed affordability ratio (AR_MHI_, AR_CP_ and AR_DP_) to minimize confounding induced by missing data potentially correlated with our outcome variables. A comparison of models first with and then without system size using the AIC criterion and an F-test indicated that the model including system size improved model fit for each of the three affordability ratios (S6 Table in S6 File). This indicates that affordability ratio estimates, across income levels, are significantly associated with system size.

Table 4 summarizes crude and adjusted means and Tukey’s post-hoc test results for each affordability ratio. Adjusted means are lower than observed means across the three affordability ratios. Further, confidence intervals indicate that our sample means are marginally outside the bounds of predicted affordability ratios, but the overall trend of ratios across system size categories remains the same. Very small and small systems had significantly higher average affordability ratios (for all income levels) compared with medium and large systems (*p* < 0.001) after controlling for potential confounders associated with missingness and affordability. Mean differences were significant in pairwise comparisons between very small or small systems (where adjusted mean AR_CP_ were 2.2% and 1.9%, respectively) and medium or large systems (where average AR_CP_ were 1.6% and 1.3%, respectively) (*p* < 0.001).

**Table 4:** Crude and adjusted mean affordability ratios by system size, for 2015^‡^.

	AR_MHI_		AR_CP_		AR_DP_	
System size (Number of people) Number of systems	Crude means (SD)	Adjusted means (95% CI)	Crude means (SD)	Adjusted means (95% CI)	Crude means (SD)	Adjusted means (95% CI)
**Very Small** (<500) *n = 661*	1.3 ± 1.1	1.0 (0.96, 1.1)^a^	2.8 ± 2.2	2.2 (2.0, 2.3)^a^	5.6 ± 4.3	4.3 (4.1, 4.5)^a^
**Small** (501–3,300) *n = 304*	1.3 ± 1.3	0.9 (0.9, 1.0)^a^	2.3 ± 1.6	1.9 (1.8, 2.1)^a^	4.5 ± 3.2	3.8 (3.6, 4.1)^a^
**Medium** (3,301–10,000) *n = 166*	0.9 ± 0.6	0.7 (0.7, 0.8)^b^	1.7 ± 0.9	1.6 (1.4, 1.7)^b^	3.4 ± 1.7	3.1 (2.8, 3.4)^b^
**Large** (10,000+) *n = 370*	0.7 ± 0.4	0.6 (0.6, 0.7)^b^	1.5 ± 0.7	1.3 (1.3, 1.5)^b^	2.9 ± 1.4	2.7 (2.5, 2.9)^b^

^‡^ Results are rounded to the tenth of a decimal for percentages. For adjusted means estimated with the *ggeffects* package in R, covariates were held to average or, for factor variables, proportional relative to sample. All data were log transformed for statistical tests and back-transformed for the table. Means that share the same letter column-wise are not significantly different from one another based on Tukey’s Honest Difference post-hoc tests on log-transformed affordability ratios in the general linear models shown in S6 Table in S6 File. Post-hoc letters are ordered descending from the highest mean.

We find that, across systems with data, poverty and deep poverty level affordability ratios increase as system size decreases. Affordability ratios for households in deep poverty follow the same trend. Households at poverty and deep poverty income levels thus have a higher water bill burden if they are in a very small or small system compared to households with similar incomes in larger systems (Table 4). Post-hoc trends held in our sensitivity analysis, but as systems with less reliable data were removed from the analysis, the differences in means by system size became larger (small systems had significantly higher mean ARs than very small average affordability ratios) for affordability ratios at county and deep poverty levels (S7 File).

### 5.4 Household poverty indices by system size

Our estimates of households in systems earning at or below the county poverty (HH_CP_) or deep poverty levels (HH_DP_) indicate the proportion of households within a system facing *at least* the associated affordability ratios. Nearly all water systems have some percentage of households living at or below the county poverty level (Table 5). The median percentage of such households in the sample is 24% (IQR = 15%; 31%) and the median percentage at or below deep poverty is 9% (IQR = 5%; 13%).

**Table 5:** Percentage of households at or below county poverty level and deep poverty level across systems in sample (n = 1501) and full community water system list with system boundaries (n = 2882), for 2015^‡^.

	Water systems in Affordability Study (n = 1501)		Full Water System List with Boundaries (n = 2882)	
System Size (People in System)	Households at or below County poverty level,	Households at or below Deep poverty level,	Households at or below County poverty level,	Households at or below Deep poverty level,
	HH_CP_ (%)	HH_DP_ (%)	HH_CP_ (%)	HH_DP_ (%)
Very small	22 ± 13^c^	9 ± 7^b^	24 ± 14^b^	10 ± 7^b^
(<500)				
Small	28 ± 14^a^	11 ± 8^a^	28 ± 14^a^	11 ± 8^a^
(501–3,300)				
Medium	25 ± 11^a,b^	10 ± 6^a^	25 ± 11^a,b^	10 ± 5^a^
(3,301–10,000)				
Large	24 ± 8^b^	10 ± 4^a^	24 ± 9^b^	10 ± 5^a^
(10,001+)				

^‡^ Results are rounded to the nearest integer. For post-hoc tests, all data were square-root transformed to ensure normally distributed residuals, and back-transformed for the table. Means that share the same letter column-wise are not significantly different from one another based on Games-Howell non-parametric post-hoc tests on square-root-transformed data. Post-hoc letters are ordered descending from the highest mean.

System size was significantly associated with household poverty indices for the full system list and for the sample list using Welch’s One-Way ANOVA (*p* < 0.001). The effect size of system size on poverty levels was very small (eta squared = 0.03 and 0.01 for sample list and full system list, respectively), indicating that differences in poverty levels across system sizes were statistically significant but somewhat marginal in absolute terms. All system sizes had an average HH_CP_ estimate greater than 20% (Table 5).

The distribution of HH_CP_ and HH_DP_ in the sample of 1,501 systems is not significantly different from that for the overall community water system list (Mann Whitney U-test *p* = 0.07). For very small systems, however, the Mann Whitney U test indicates significant differences in HH_CP_ and HH_DP_ between the sample and full water system list (*p* < 0.001). Mean poverty levels for very small systems are lower in the sample (Table 5), with means between the sample and full list differing by around 2% (Table A in S5 File). This corroborates the 5% underrepresentation of low-income systems in our bias assessment. Given this and the directionality of the trends we identified where affordability ratios are higher in smaller systems, it is likely the sample under-estimates the magnitude of smaller systems with affordability problems.

**Fig 2:**
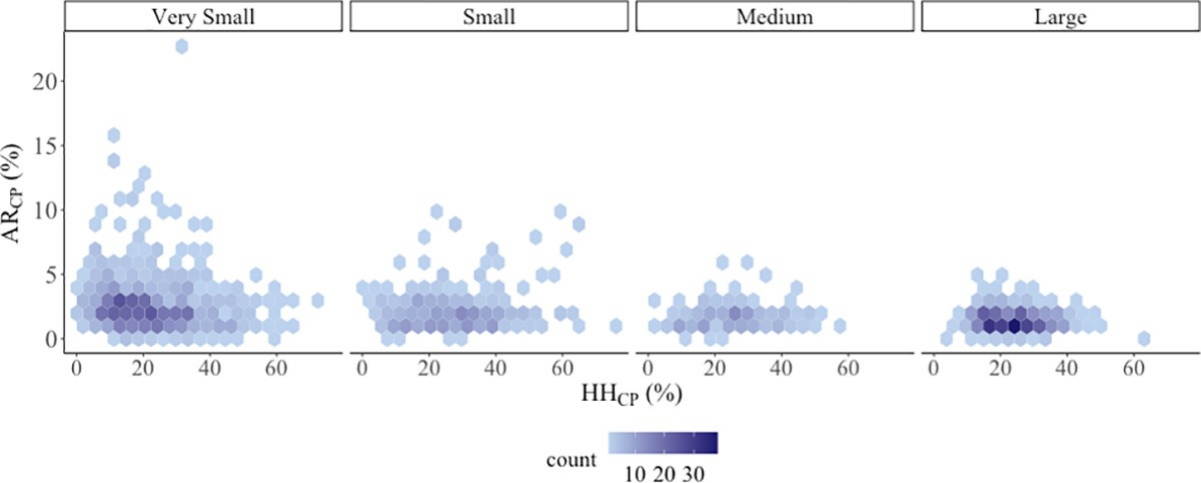
Percentage of households below county poverty threshold (HH_CP_) vs. affordability ratio at county poverty threshold (AR_CP_), by community water system size (n = 1,501). Color fill shows the number of systems in each hexagon. Strip text above plots indicate community water system size by population served: very small = 25–500 people; small = 501–3,300 people; medium = 3,301–10,000 people; large = 10,000+ people.

Fig 2 shows the relationship between poverty-level affordability ratios (AR_CP_) and the percentage of households in poverty within a system (HH_CP_). The figure can be used to assess the prevalence of systems at various affordability ratios and poverty levels, while keeping in mind the underrepresentation of very small systems. For example, across system sizes, we see many systems with greater than average poverty levels, but relatively affordable water (indicated where AR_CP_ is less than 1–2%). However, a fifth of all water systems (n = 318) were estimated to have at least a third of households in poverty (i.e. HH_CP_ = 33%). For these 318 systems, the average AR_CP_ was 2.1%. This signals potential unaffordability for households in poverty (especially were sewer to be added), and vulnerability for a substantial fraction of systems (21%) whose customer base was economically vulnerable. Fig 2 also shows that some systems–usually small or very small–had high percentages of households at or below the county poverty level (e.g. HH_CP_ > ~10%) and relatively unaffordable water bills (e.g. AR_CP_ > ~3%).

## 6 Discussion and conclusion

We developed a key-dimensions based approach to measuring water affordability in a human right to water context and applied it to the case of California’s community water systems. This study offers four contributions to water affordability research. First, we develop affordability measures using publicly available data by conceptually linking measurement and data choices to broader aims for the human right to water. We demonstrate how these dimensions can be applied as broad guidelines to develop transparent affordability measures. Future studies can build on and use these dimensions as a lens to assess and evaluate their data and methods. These dimensions might also serve as the basis for more sensitivity analyses among different measure choices. Second, we develop two new California-specific water affordability ratios for households in poverty and improve on median income affordability ratios by focusing on basic needs water use. While poverty here is specific to California counties, other locally-specific poverty levels could be substituted in studies seeking to develop a similar set measures in other contexts. Third, our analysis is, to our knowledge, the first statewide assessment of water affordability for households at different income levels served by community water systems in California, and one of the few studies that includes very small systems. While the underrepresentation of very small systems in our sample prevents us from a definitive conclusion on the state of water affordability, we show how publicly-available data can be used to identify trends and areas for future investigation. We innovate on past affordability research by developing multiple ratios for a policy context, rather than adhering to a single income-level or threshold of affordability. The ultimate determination of affordable / unaffordable is–in the end–a political decision for local contexts, towards which the ratios we developed can contribute. Finally, we co-produced this research with a government agency that develops tools to track and monitor California’s human right to water. Co-production as a mode of scientific research enhances the relevance of research questions and methods while also increasing the likelihood of research translation into decision-making [72].

Collectively, we find that water was relatively affordable for households earning at median income levels in a majority of California water systems for which 2015 data were available. Across these systems, average AR_MHI_ equals 1.1% without the explicit inclusion of sewerage. Despite known critiques of AR_MHI_ as a measure, we nonetheless argue that it is a useful complement to poverty-focused metrics, as it describes impacts for 50% of households and can signal affordability risks in water systems where a majority of households are low-income. For instance, we identified several cases where a water system’s MHI was lower than the county poverty threshold. For the 41 systems in which median household incomes were below their county poverty thresholds, the average affordability ratio was nearly three times the state-wide average (3.1%).

Our two poverty-focused affordability ratios show that the median county poverty level affordability ratios were around the unaffordability threshold as defined by California’s 1.5% thresholds for median income levels in low-income systems (median AR_CP_ = 1.8%). Water was substantially unaffordable for households earning at the deep poverty level. In particular, a quarter of water systems had average bills at 2.7% of county poverty level incomes and 5.3% of deep poverty levels. For economically vulnerable households, even average water bills can be unaffordable. Moreover, households at the poverty level in systems with the highest affordability ratios (i.e. the top 10^th^ percentile) had water bills that were 2.4 times the state-wide average, or $125.10 per month for 6 HCF. Such cases indicate that affordability is not driven just by low incomes but also very high water bills. These affordability ratios for economically vulnerable households are cause for concern because water rates, especially in urban areas, have been rising faster than inflation [14] while incomes have stagnated or declined for middle and low-wage earners [73, 74].

Our study also underscores the impact of unaffordable water bills on smaller systems. On average, affordability ratios were highest for households served by very small and small systems at every income level evaluated in this study. This is in part because households served by smaller systems (25–3,300 people) had some of the highest monthly water bills for 6 HCF. Yet even within larger water systems and systems categorized as non-disadvantaged, affordability ratios for households at the county and deep poverty income levels were often close to, or exceeded, international and national thresholds for affordability.

The results and methodological contributions highlight several policy implications. First, our findings reveal heterogeneity in affordability challenges for households across and within water systems in the state and suggest that affordability support should not overlook household-level struggles. Until recently, current state-wide approaches to ensure affordable water have focused on economically disadvantaged systems whose upgrades to comply with water quality standards will push water bills above 1.5% of the water system’s median household income [34]. Providing financial resources to economically disadvantaged systems is critical, but households also struggle to pay for water in larger, non-disadvantaged CWSs. Though many of these larger systems may provide direct subsidies, households are under-enrolled and policies vary across systems [7, 33]. This point is echoed by other work that notes the prevalence of poverty in large, urban water systems [25, 27, 30, 75].

Second, as discussed above, we assess affordability for households consuming 6 HCF per month in an effort to exclude luxury water consumption that is not protected by a human rights framework. This is defensible by ‘basic needs’ water requirements and conservation standards in California. Nonetheless, households may need higher water volumes for even basic needs in certain contexts (e.g., households with many members or sick members). Thus water affordability implementation programs may wish to cast a wider safety net for affordability.

Our study addresses several critiques of commonly-used affordability ratios, but there remain areas for continued research and improvement. First, just over half of the California community water systems (1,501 out of 2,901 community water systems) had adequate data to evaluate water affordability. Our study found an under-representation of very small systems with affordability data by 18.5% (S5 File). Most of the missing data was a result of systems not reporting water bills. Incomplete data is a substantial barrier to comprehensively tracking the human right to water and can lead to bias in conclusions about affordability. We thus incorporated predictors of missingness in our model of affordability by system size in attempt to reduce biased inferences.

Together, these data limitations indicate broader trends in research where smaller or rural systems with poor data are frequently underrepresented [76]. While our study could have excluded these systems to solely look at the state of medium to large systems affordability, the human right to water compels state agencies to provide as much representation as possible for *all* communities. Our interpretations are tempered by the lack of a fully representative sample. As this study is meant to offer an approach to using publicly available data, however, we believe that excluding the 661 very small systems with data for the sake of statistical accuracy would have sharply limited our understanding of where the state needs to drive data collection efforts and where affordability challenges are–in our assessment–worst. This approach outlines two inter-related paths moving forward: 1) agencies ought to characterize missingness in their evaluations while designing solutions–such as randomized sampling efforts–to collect a representative sample of systems to overcome bias from missing data, and 2) research must increasingly work to fill these gaps in order to better track the realization of the human right to water.

Secondly, while we assessed data reliability and applied sensitivity analysis to data that we flagged as unreliable due to high sampling error in the American Community Survey, we did so only for water systems falling within one block group (504 systems of the 1,501 in the study). For the remaining systems, we aggregated block group level data to the water system service area. Our approach used population and household weighted areal spatial aggregation, enabling better representation within water systems. County poverty levels for bigger rural counties were grouped together due to census error, and while they still represent poverty-level incomes, their income levels likely bias against accurate data for smaller, rural water systems. More research is needed to assess data reliability of census estimates in new geographies that do not overlap with census boundaries.

Thirdly, the income constraints across affordability ratios are estimates that should be interpreted with care. Median income levels do not account for taxes or other non-elective expenditures, and so affordability ratios based on these underestimate the potential problem for median-income households. California poverty thresholds are limited in that they do not address inter-county heterogeneity. These income levels could underestimate disposable incomes for households because they do not include benefits such as housing subsidies, but may also overestimate real disposable income to the extent that expenses for non-water essentials are not removed.

Finally, we hypothesize that our findings somewhat underestimates the affordability challenge in California for two reasons. First, our sample had 5% fewer very-low income systems (less than 60% state MHI) represented (S5 File). Second, inclusion of stormwater or sewer rates would indicate that our results underestimate the challenge. A 2015 state survey of residential sewer rates with data for 435 agencies shows a median sewer charge of $37.00 per month [77]. This data was not incorporated in our analysis because we could not determine which of the 2,901 CWS from the eAR survey had sewer services, and therefore we could not evaluate the representativeness of this smaller survey sample.

In addition to improving upon these methodological challenges, research is needed to capture a broader range of affordability concerns. Examples include assessing full costs, e.g. connection fees [40] or well maintenance costs, and tracking water shut-off consequent to the lack of means [1, 13]. Populations that do not have representation in current affordability ratios [18, 60, 78] or in this study require greater focus. These include people without homes, mobile home park residents, households served by systems with fewer than 15 connections (“state smalls”), and private well-owners. Furthermore, the impact on households forced to buy bottled water due to poor tap water quality are not factored into this study. Previous work has shown that increased water costs, the risk of poor water quality, low water-system financial capacity, and high concentrations of low-income communities of color–particularly in unincorporated communities–are entwined [15, 79]. Community mistrust in tap water is also a driver of bottled water consumption, with the resultant time and money costs falling disproportionately on communities of color [80]. These multidimensional aspects of affordability demand better data, additional metrics, and increased representation of marginalized groups in human right to water monitoring efforts.

Future research goals for this work therefore include investigating the extent to which household water affordability relates to ethnic or racial disparities and to human right to water pillars such water quality and accessibility. Moreover, the findings here are absent sewer charges which are often greater than drinking water bills and likely to worsen the outlook of the assessment presented here. Measures play a central role in representing water affordability, underscoring the importance of ongoing debate on what we can, and should, measure to ensure new policies equitably realize the human right to water in California and beyond.

## Supporting information

S1 File Data sources & data cleaning overview. (PDF)

S2 File Sensitivity analysis–water bills. (PDF)

S3 File Areal-household weighting methodology. (PDF)

S4 File Sensitivity analysis–missing or incomplete data. (PDF)

S5 File Bias assessment and measured confounding. (PDF)

S6 File F-test for affordability ratio models with and without system size. (PDF)

S7 File Sensitivity analysis results. (PDF)

S8 File Affordability ratios by common affordability thresholds. (PDF)
