# Supplementary material for: Water affordability and human right to water implications in California
Source: PLoS One. 2021 Jan 20;16(1):e0245237. doi: 10.1371/journal.pone.0245237 (PMC7816992; doi:10.1371/journal.pone.0245237)
Supplement: S1 File — (PDF) [file pone.0245237.s001.pdf]

Water affordability and human right to water implications in California

Jessica J. Goddard[1,2], Isha Ray[1], Carolina L. Balazs[2]
[1] Energy & Resources Group, University of California, Berkeley, California, United States of America
[2] Office of Environmental Health Hazard Assessment, California Environmental Protection Agency, Oakland, California

S1 Text. Data Sources & Data Cleaning Overview

Systems from the State Water Board's electronic Annual Review database from 2015 were included in the study if they met the following criteria:

1) Water system answered the survey question: "Average monthly residential customer water bill in $/month using 6 CCF; 12 CCF; 24 CCF" for the 6 hundred cubic feet level *and*,
2) Water system did not answer the survey question but reported a Flat Base Rate (FBR) billing structure:
    a) *and* a monthly billing frequency *or* an explicit billing frequency in notes (e.g. "per quarter")
    b) *and* no other prices were reported in non-FBR rate categories.

Large system survey: *https://drinc.ca.gov/ear/EARFromTable.aspx?SurveyID=15&SectionID=6*

Small system survey: *https://drinc.ca.gov/ear/EARFromTable.aspx?SurveyID=16&SectionID=6*

Systems were excluded from the study if they met the following criteria:

1) Water system was not in community water system list obtained from the Office of Environmental Health Hazard Assessment (n = 2,901 community water systems);
2) Water system reported rates instead of monthly water cost, demonstrated by looking at a system's reported rate structure and reported rate prices;
3) Water system had clear reporting error;
4) Water system had no median household income data.

Systems were removed from the analysis of affordability ratios by system size in a sensitivity analysis if they met the following criteria:

1) Water system was above or below the upper and lower fences of our outlier assessment (See S4);
2) Water system had over 15% of a system's aerially-weighted households had no available income data (See S3)
3) Water system was within one block group and the Median Household Income estimate was considered unreliable in an analysis of Census sampling error (See S4 below).

Systems were removed from the study of household poverty indices by system size in a sensitivity analysis if they met the following criteria:

1) Water system was within one block group and Households within Income bracket data from Census was considered unreliable in an analysis of Census sampling error (See S4);

S1 Figure A demonstrates the results of this data cleaning, exclusion, and processing with counts of water systems excluded from raw data.

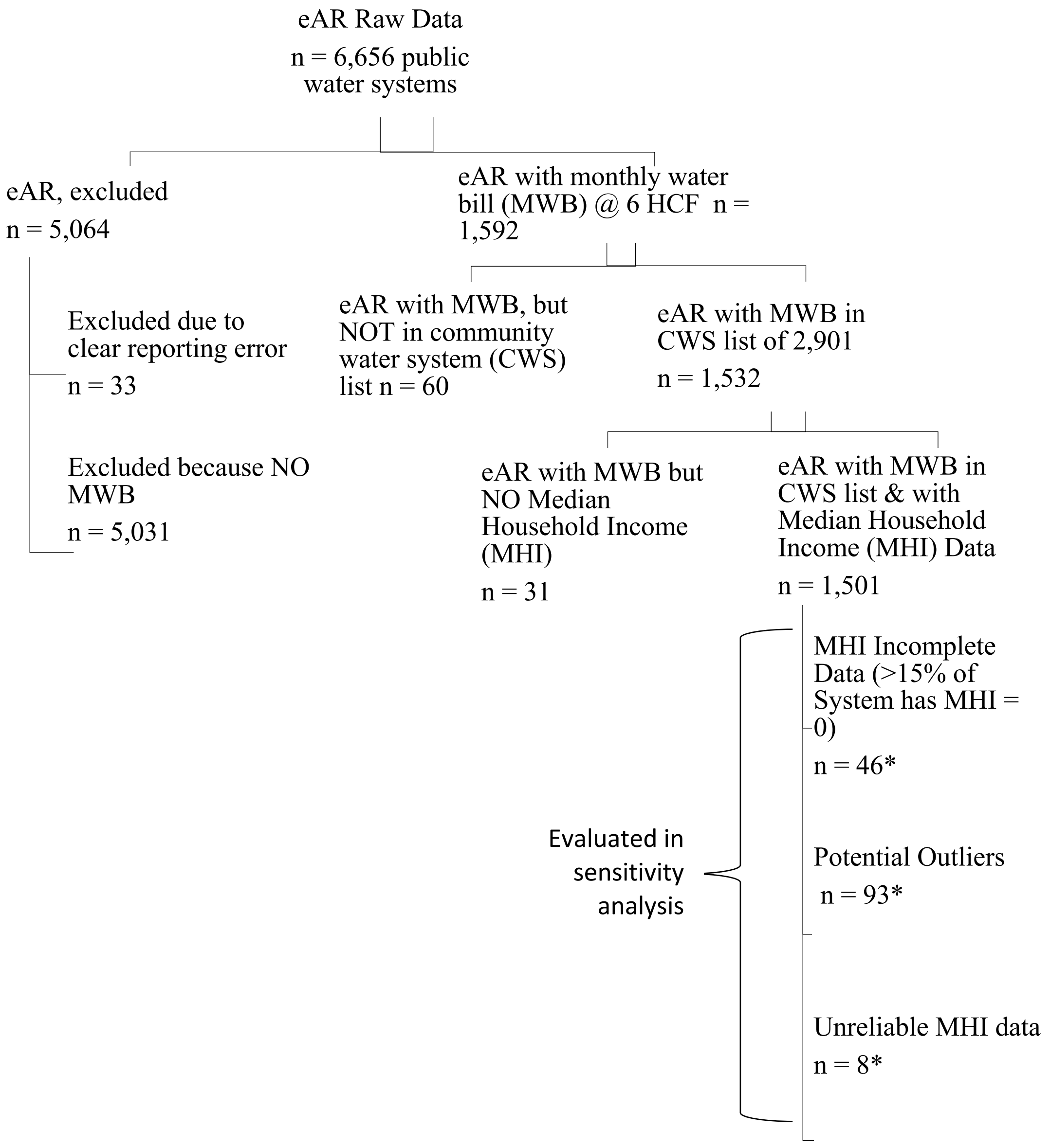

*Three systems overlap between outlier systems and MHI incomplete data.

S1 Figure A. Data cleaning for water bills.

S1 Table. Data sources used to create affordability measures and household poverty indices.

Data Type	Data Source	Available:	Manipulation to Match to Water System Boundaries	Excluded in a sensitivity analysis if:	Potential Sources of Error
Water Bills	State Water Resources Control Board electronic Annual Reports (eAR) 2015	Original data retrieved from Division of Drinking Water in 2016 from: https://drinc.ca.gov/drinc/DWPRepository.aspx<br><br>Recent data available: https://www.waterboards.ca.gov/drinking_water/certlic/drinkingwater/ear.html	None	Water bill >= $177.32 or <= $13.91 (See S2)	Reporting error or non-response
Median Household Income	ACS Census 2011-2015 5-Year Estimates (Formerly Table B19013; now S1901)	https://data.census.gov/cedsci/	Areal-household weighting using water system and block group boundaries (See S2)	Systems that have 15% or more of block groups with missing MHI data (see S4)<br><br>System is within one block group and has unreliable Census estimate (see S4)	Estimate for water system is not 'true' median but an average of underlying median incomes<br><br>Assumption of geographic homogeneity in block groups (error increases for more rural geographies)<br><br>Census data reliability
California Poverty Income Threshold	PPIC California Poverty by County (2015)	https://www.ppic.org/map/california-poverty-by-county-and-legislative-district/<br><br>County poverty thresholds weighted by number of renters and owners in 2015 retrieved from PPIC.	Water system assigned county poverty income and deep poverty income based on county	None	Of the 58 counties, 38 counties have unique thresholds and the remaining 20 are in 3 groups due to Census suppression criteria [1]
Households by Income Bracket & Total Households	ACS Census 2011-2015 5-Year Estimates (Formerly Table B19001; now S1901)	https://data.census.gov/cedsci/	Areal-household weighting using water system and block group boundaries (See S2)	Water systems with more than one estimate (for Total Households data) or more than 20% of estimates (for Households by Income Bracket) have unreliable Census estimates (see S4)	Assumption of geographic homogeneity in Census Tracts (error increases for more rural geographies)<br>Census data reliability (i.e. sampling error)

S1 References

1. Bohn S, Danielson C, Levin M, Mattingly M, Wimer C. Technical Appendices: The California Poverty Measure: A New Look at the Social Safety Net. 2013. Available: https://www.ppic.org/content/pubs/other/1013SBR_appendix.pdf
