# Supplementary material for: Water affordability and human right to water implications in California
Source: PLoS One. 2021 Jan 20;16(1):e0245237. doi: 10.1371/journal.pone.0245237 (PMC7816992; doi:10.1371/journal.pone.0245237)
Supplement: S2 File — (PDF) [file pone.0245237.s002.pdf]

PLOS ONE

Water affordability and human right to water implications in California

Jessica J. Goddard[1,2], Isha Ray[1], Carolina L. Balazs[2]
[1] Energy & Resources Group, University of California, Berkeley, California, United States of America
[2] Office of Environmental Health Hazard Assessment, California Environmental Protection Agency, Oakland, California

S2 Text. Sensitivity Analysis–Water Bills
Given the extreme range of reported values in the eAR survey for water bills across various volumes (i.e. from $3.06 to $466 per month for 6HCF), we conducted a sensitivity analysis, analyzing the results of the study with and without systems with potentially extreme water bill values. The complex nature of water system ownership and heterogeneity among rate structures in California makes a qualitative prior for thresholds challenging to determine. Therefore, we used cut-off points determined from adjusted box plots that account for distribution skew [1]. The upper and lower fences determined by this method served as benchmarks to explore very high and very low water bill values given no prior systematic evaluation of eAR survey data at a fixed volume. It is important to note that the water systems falling above or below the threshold set by the Hubert & Vandervieren (2008) method are statistical outliers, not necessarily real outliers. This results in a conservative approach toward assessing sensitivity in the eAR data.

Of the 1,532 community water systems with useable water bill data (regardless of available income data), 29 systems fell above the upper fence of $181.78, and 69 system fell below the lower fence of $14.79, for a total of 98 potential outlier systems (Table S2).

S2 Table A. Identification of upper and lower thresholds used to exclude outliers in sensitivity analysis.

**Metrics**	**Results***
Q1	$29.29
Median of Dataset	$41.56
Q3	$61.60
Interquartile Range (IQR)	$32.32
Medcouple (MC) +	0.3
Lower Fence (threshold) = Q1 – [1.5 × exp(-4 × MC) × IQR]	$13.91
**Number of systems below lower fence**	**69**
Upper Fence (threshold) = Q3 + [1.5 × exp(3 × MC) × IQR]	$177.32
**Number of systems above upper fence**	**29**

*All calculations were conducted using adjboxStats in the robustbase package of R 3.5.1 [2]
+The medcouple is the median of an array calculated using the kernel function as reported in the adjusted box plot method. A positive value (MC > 0) reflects a right-skewed distribution.

Systems above and below the thresholds were cross-listed with a survey of extremely high and low water bills that we conducted in 2015 as part of the Office of Environmental Health Hazard Assessment (OEHHA) Human Right to Water project. At the time, we investigated the reliability of extreme water bills based on upper and lower fences determined given bills for 12 hundred cubic feet (HCF) of water. We asked water systems to answer the same question posted on the Water Board's electronic annual report survey about rate data and to estimate water bills at 6, 12, and 24 hundred cubic feet. Systems were contacted three times by phone and email before being labeled 'unreached.' While we also collected data for 6 HCF water bills for very low and very high water bills, the survey is not directly representative of the systems flagged in this outlier assessment because the original sample was drawn from the 12 HCF water bill data. However, of the 98 systems with affordability data flagged in this study's outlier assessment (as having water bills over $177.32 and below $13.91), 86 were contacted as part of the 12 HCF survey. Of these 86 systems, 37 (43%) responded to the survey.

S2 Figure A compares the water system response to the Berkeley survey question (y-axis) about water bills for 6 HCF versus the eAR survey question (x-axis) for low-outliers and high outliers, respectively. As expected, many systems reporting very low water bills reported higher water bills for 6HCF in the phone survey. The reverse held true for systems responding about potentially very high water bills. Nevertheless, several systems with very low and very high water bills were in fact accurate. These results supported our choice to exclude systems with very low or very high water bills in a sensitivity analysis to determine the effect on our outcomes in the affordability ratio study.

S2 Figure A. Results of water system survey to investigate very low and very high water bills.
Original sample design included 86 of the 98 water system flagged as potential outliers in the present study. Red dashed line represents the line of equality between the Berkeley and eAR survey.

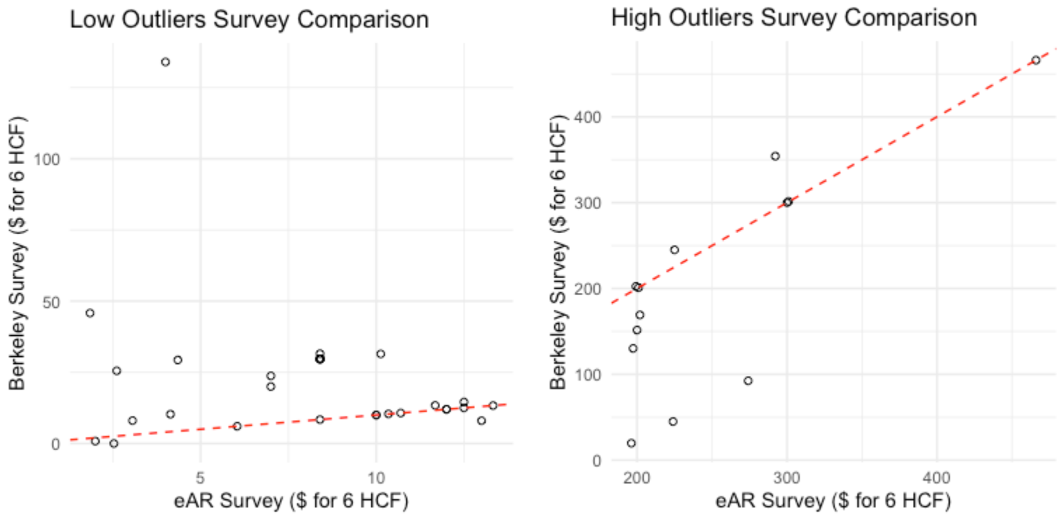

S2 References

1. Hubert M, Vandervieren E. An adjusted boxplot for skewed distributions. Comput Stat Data Anal. 2008;52: 5186–5201. doi:10.1016/j.csda.2007.11.008
2. R Core Team. R: A language and environment for statistical computing. Vienna, Austria: R Foundation for Statistical Computing; 2018. Available: https://www.r-project.org/
