# Supplementary material for: Water affordability and human right to water implications in California
Source: PLoS One. 2021 Jan 20;16(1):e0245237. doi: 10.1371/journal.pone.0245237 (PMC7816992; doi:10.1371/journal.pone.0245237)
Supplement: S3 File — (PDF) [file pone.0245237.s003.pdf]

PLOS ONE

Water affordability and human right to water implications in California

Jessica J. Goddard[1,2], Isha Ray[1], Carolina L. Balazs[2]
[1] Energy & Resources Group, University of California, Berkeley, California, United States of America
[2] Office of Environmental Health Hazard Assessment, California Environmental Protection Agency, Oakland, California

S3 Text. Areal-Household Weighting Methodology

Given a water system boundary that spans several census blocks and census block groups, the following approach was used to attribute census data used in the study from the American Community Survey to water system boundaries. Below, the methodology is demonstrated for use with median household income data, though the same approach applies to other census data in the study (number of households and number of households within income levels).

Intersection of populated blocks with water system boundaries was conducted using ArcGIS 3.0 at OEHHA (see description below). Block level populated household data and shapefiles were obtained from the 2010 Census (Available for download: https://www.census.gov/geo/maps-data/data/tiger-line.html) and overlaid with water system boundaries from the Tracking California website (https://trackingcalifornia.org/water/map-viewer). Water system boundaries were adjusted according to methods published for CalEnviroScreen 3.0 [1].

Let:
Block = i
Block Group= j
Block within a Block Group = ij
Populated household = HH
Area of water system = A_x
Area of block = A_i
Area of block in water system x = A_{xi}

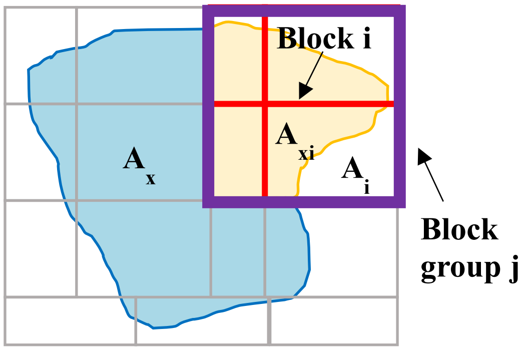

In order to calculate the median household income (MHI) of a given water system (x):
The number of households in block i contained within a block group j contributing to a water system x's area is determined by:

$$HH_{xij} = HH_{ij} \times A_{xi}$$

Where, the A_{xi} is the proportion of block i in the area of the system (A_x):

$$A_{xi} = w_x A_i$$

Where w_x is the aerially determined weight (or the areal difference as a percentage from ArcGIS). The sum of households from blocks i is thus equal to the total households in block group j (BG_j) that intersect with the water system:

$$\sum_{i=1}^{i=n} HH_{xij} = HH_{xj}$$

HH_{xj} is aerially apportioned sum of households associated with block group j in system x. Note: Summing across all block group j would result in an estimate of the total number of households for water system x (HH_x):

$$\sum_{j=1}^{j=n} HH_{xj} = HH_x$$

To obtain the median household income of water system x, we first multiply the MHI associated with block group j by the adjusted household sum for block group j (HH_{xj}) and sum over all

block groups associated with system x. Then we divide this sum by the total number of households for the system x:

$$MHI_x = \frac{\sum_{j=1}^{j=n}(MHI_j \times HH_{xj})}{\sum_{j=1}^{j=n} HH_x}$$

For systems flagged as potential unreliable due to missing data in block groups (See B4 below), water system estimates of MHI were calculated excluding the adjusted household sum (HH_{xj}) contributions from block groups with missing data. To calculate the number of households within each income bracket within a water system, we applied the same methodology. To estimate social demographic data used in the study of missing data, we applied the same methodology but used population counts rather than household counts.

S3 References

1. OEHHA. Methodology for a Statewide Drinking Water Contaminant Indicator, CalEnviroScreen 3.0. 2017 [cited 27 Mar 2019]. Available: https://oehha.ca.gov/media/downloads/calenviroscreen/report/ces3dwmethodology.pdf
