# Supplementary material for: Water affordability and human right to water implications in California
Source: PLoS One. 2021 Jan 20;16(1):e0245237. doi: 10.1371/journal.pone.0245237 (PMC7816992; doi:10.1371/journal.pone.0245237)
Supplement: S4 File — (PDF) [file pone.0245237.s004.pdf]

PLOS ONE

Water affordability and human right to water implications in California

Jessica J. Goddard[1,2], Isha Ray[1], Carolina L. Balazs[2]
[1] Energy & Resources Group, University of California, Berkeley, California, United States of America
[2] Office of Environmental Health Hazard Assessment, California Environmental Protection Agency, Oakland, California

S4 Text. Sensitivity Analysis–Missing or Incomplete Data

Median household income (MHI) data were downloaded from the American Community Survey (ACS) 5-Year Table B19013 at the block group scale. Of the 1,532 water systems with water bill data, 31 systems had block groups with no MHI data. Of the 1,501 water systems with water bill, 342 systems contained or overlapped with block groups that had missing MHI data. Of these 342 systems, 46 systems had missing MHI data for more than 15% of the households, as determined by the aerially weighted household contribution of the block group to the water system. These systems were flagged as potentially unreliable for having high amounts of missing data. No data was missing or incomplete for the total number of households or households across income brackets (ACS 5-Year Table B19001).

Census income data reliability

While the ACS already controls for error before publishing results, they provide quantitative information on sample error for their estimates. This is effectively a measure of estimate imprecision. The ACS provides margin of errors (MOE) at 90% confidence levels by quantifying the variance and standard errors of the estimates resulting from the sampling approach using a successive differences replication (SDR) variance estimation methodology [1,2]. From the variance, ACS calculates the standard error (square root of the variance) and the margin of error at a 90% confidence level:

$$Standard\ Error = \sqrt{Variance}$$

$$Margin\ of\ Error_{90\%\ Confidence\ Interval} = Standard\ Error \times 1.645$$

Given the census provided margin of error estimates, we can back-calculate standard errors to estimate coefficients of variation. The coefficient of variation (COV), equivalent to the relative standard error, measures the ratio between an estimate's standard error and the estimate itself:

$$Coefficient\ of\ Variation = \frac{Standard\ Error}{Estimate} \times 100$$

Coefficients of variation are then used to determine 'reliability' of data points.

Reliability criteria using coefficients of variation

Determining what constitutes a reliable data point based on COVs is not a clear cut decision. Furthermore, for income estimates at the water system scale, we use an areal-household weighting approach to arrive a weighted-average estimate for each water system. To our knowledge, no precedent exists for calculating new MOEs at aggregate geographies that are not simply additive or multiplicative changes within census boundaries. The latest research on census data geography aggregation for error minimization employs a sophisticated algorithm to improve aggregation techniques, but even here the authors work within given census geographies [3]. As such, we develop criteria to flag potentially unreliable census estimates for systems falling within one block group.

We use three sets of estimates: median household income, households by income bracket (16 brackets), and total number of households. Median household income data is used for the affordability ratio at the median household income level (AR_{MHI}). Total households and the number of households in each income bracket are used for creating household indices of poverty

in the study (HH_{CP} and HH_{DP}). We use the following exclusion criteria to evaluate census data, as outlined in CalEnviroScreen 3.0 [4] for census tracts; in this case, we use block groups:

a) Coefficient of variation greater than 50 (meaning the standard error was less than half of the estimate) and,
b) Standard error was greater than the mean standard error of all California census block groups estimates for the data of interest.

In cases where coefficient of variation is incalculable (e.g. an estimate is 0), we can only look at whether the standard error (MOE/z_{90}) is less than the average, per the second half of the inclusion criteria. We assume that if the standard error is less than the mean of all block groups, the estimate is reliable by the criteria which we can measure.

The household poverty indices (e.g. HH_{CP} and HH_{DP}) estimated for each system are created by summing the number of households below a specified income level; linear interpolation is used between estimates if the income level falls within the census brackets (e.g. between $20,000-$25,000). As such, the estimated household index is only impacted by an unreliable estimate if the household index encompasses an unreliable estimate. The unreliable estimate may not impact the interpolation at all. We thus chose a broader criteria and excluded systems in the sensitivity analysis if more than 20% of the estimates were unreliable by our criteria.

Results of reliability study for sensitivity analysis

Coefficients of variation for census estimates for systems within one block group

Of the 1,501 systems with water bill and income data, 505 systems fall within one block group. Of the 505 systems with one block group, 429 of them (85%) have fewer than 200 connections. Of systems with only one block group (505 systems), there are 9,090 total estimates to evaluate (18 estimates for each system).

Total households and Median household income estimates. There are no estimates in the total household data that meet the unreliability criteria. Of the 505 systems with data to evaluate reliability, there is one median household income estimate with no margin of error (and thus no coefficient of variation could be calculated). Of the 504 water systems with estimates for median household income, 8 systems had unreliable estimates.

Households by income bracket. Of the 505 systems, 248 systems had 20% of their more than 2 unreliable estimates among the 16 households by income bracket estimates. Of these 248, 5 systems overlapped with the 8 systems found to have unreliable median household income estimates.

Final list systems to exclude in a sensitivity analysis. Of the 1,501 systems with income and water bill data, 8 systems were excluded from the affordability assessment in a sensitivity analysis. Of the 1,501 systems for which we estimated household poverty indices by system size, 227 systems were excluded in a sensitivity analysis.

S4 References

1. U.S. Census Bureau. American Community Survey Multiyear Accuracy of the Data (5-year 2011-2015). 2015. Available: https://www2.census.gov/programs-surveys/acs/tech_docs/accuracy/MultiyearACSAccuracyofData2015.pdf?#
2. U.S. Census Bureau. Documentation for the 2011-2015 Variance Replicate Estimates Table. 2014. Available: https://www2.census.gov/programs-surveys/acs/replicate_estimates/2015/documentation/5-year/2011_2015_Variance_Replicate_Tables_Documentation.pdf
3. Spielman SE, Folch DC. Reducing Uncertainty in the American Community Survey through Data-Driven Regionalization. PLoS ONE. 27 Feb 201510: e0115626. doi:10.1371/journal.pone.0115626
4. OEHHA. CalEnviroScreen 3.0. 2017 Jan. Available: https://oehha.ca.gov/media/downloads/calenviroscreen/report/ces3report.pdf
