# Supplementary material for: Water affordability and human right to water implications in California
Source: PLoS One. 2021 Jan 20;16(1):e0245237. doi: 10.1371/journal.pone.0245237 (PMC7816992; doi:10.1371/journal.pone.0245237)
Supplement: S5 File — (PDF) [file pone.0245237.s005.pdf]

PLOS ONE

Water affordability and human right to water implications in California

Jessica J. Goddard[1,2], Isha Ray[1], Carolina L. Balazs[2]
[1] Energy & Resources Group, University of California, Berkeley, California, United States of America
[2] Office of Environmental Health Hazard Assessment, California Environmental Protection Agency, Oakland, California

S5 Text. Bias Assessment and Measured Confounding

S5 Table A provides a simple bias assessment to convey the impact of missing data on the overall study. The columns in white reflect the total list of community water systems and the list of systems in the final study. The columns in the gray reflect all missing systems (for any reason, including no data or inadequate data) and systems that were excluded in a sensitivity analysis based on census unreliability (S4), and based on the outlier assessment (S2). As described in the main chapter, the system list was divided into system size categories and within each size category, measured confounders were analyzed for marginal effects on whether or not a system had missing data. This section describes the bias assessment presented in S5 Table A and expands on findings from the study of measured confounding from missingness (S5 Table B).

System size

System size categories were: Very small (<500 people); Small (501-3,300 people); Intermediate (3,301-10,000 people); Large (10,000+ people). Overall, very small systems are under-represented in the study. We see the effects of this bias in the overall list of systems with missing data (n=1,400)–a disproportionate number of smaller systems do not have data (S5 Table A). Very small systems make up about 62% of the full community water system list but 44% of the sample list. The distribution of system characteristics by size is relatively similar between the full community water system list and the systems excluded in a sensitivity analysis due to the water bill outlier criteria, with a slight bias toward excluding disproportionately more medium and large systems in the sensitivity assessment.

Income status

Per California's Water Code definition of disadvantaged community (DAC) status, a system is considered DAC if its median household income is at or below 80% of the state's median, and a severely disadvantaged community (SDAC) if its MHI is at or below 60% of that for the state (Cal. Wat. Code §79505.5 and §13476). For the year of our analysis (2015), MHI across California water systems was $61,818, making SDACs those communities with median incomes below $38,700, and DACs those communities with incomes below $51,600 but above $38,700.

Table A shows that the final study list (n=1,501) has a slightly lower percentage of systems (13.5%) characterized as severely disadvantaged (systems with a median household income less than 60% the state of California's MHI for 2015) than the overall community water system list (nearly 18.5%).

Ownership/Governance

There are five ownership categories associated with water systems, but we collapsed these to public versus privately owned systems due to low counts in some ownership category types (S5 Table A). The final study list (n=1,501) has a higher proportion of public systems relative to private systems (S5 Table A).

Region

There are eight hydrologic regions that the State Water Board and OEHHA use to designate water system region: Northern California, the Bay Area, the Eastern Sierras, Northern Sierras, San Joaquin Valley, Central Coast, Los Angeles/Southern

California, and Inland Empire/Imperial Valley. Overall, systems in Northern California, Northern Sierra, and San Joaquin Valley are slight underrepresented relative to other regions in the final study list (S5 Table A). However, systems from Northern California are more likely to be missing only among small systems (S5 Table B).

Social-demographic data
There is not evidence to suggest that communities that are poorer or wealthier, or those with higher percentages of people identifying with non-White race/ethnicities, would have a higher likelihood of water system reporting data. Nonetheless, significant disparities by race/ethnicity have been identified in water system violations, and therefore we investigated whether missing data is also correlated with poverty and/or race/ethnicity. Estimates of percentage of households identifying as all non-White race/ethnicity categories in the American Community Survey were summed to estimate Percent People of Color (POC) for each block group, following the approach outlined in S3. % POC was aerially assigned to water system boundaries and population weighted to estimate a % POC by water system. Similarly, the % of households renting and % of households under two times the federal poverty level were aerially and household-weighted to estimate water system level estimates of % Renters and % Under 2X Poverty within each water system.

For % POC, the difference in means between the full system list (n=2,882 with estimates) and the final study list (n=1,501) was significant in a two-way Mann-Whitney U ranked sum difference test ($p = 0.03$). However, absolute means and standard deviations between systems with and without affordability data were not substantial (S5 Table A). Systems without affordability data have a slightly lower estimated % POC relative to systems with affordability data. Mann-Whitney U tests between systems with and without data are non-significant for % Renters or % Under Twice the Federal Poverty Level.

For very small systems and intermediate systems, the odds of a system having missing data increased marginally for each unit increase in % POC (OR = 1.01, $p < 0.001$; S5 Table B). For very small, intermediate, and large systems, the odds of a system having missing data increased marginally for each unit increase in % Renters (OR = 1.01, 1.02, and 1.02 respectively; S5 Table B). For very small systems, the odds of a system having missing data increased marginally for each unit increase in % Under 2X Poverty (OR = 1.01, $p < 0.001$; S5 Table B). Social-demographic data does not appear to have a substantial marginal effect on whether or not a system is missing affordability data when stratified by system size. This is somewhat consistent with the fact that a small portion of systems with missing data are missing income-based estimates; rather most missing data is non-reported water bills.

S5 Table A. Assessment of bias for systems with missing data for affordability assessment.

	**List of all community water systems**		**List of systems in final study list**		**Systems in community water system list with missing affordability data**		**Systems in community water system list that did not report water bills**	
	N = 2,901	***% of Systems***	***N = 1,501***	***% of Systems***	***N=1,400***	***% of Systems***	***N = 1,369***	***% of Systems***
<500 people	1,812	62.5	661	44.0	1,151	82.2	1,121	81.9
501-3,300 people	447	15.4	304	20.3	143	10.2	142	10.4
3,301-10,000 people	224	7.7	166	11.1	58	1.1	58	4.2
10,000+ people	418	14.4	370	24.6	48	3.4	48	3.5
	N = 2,901	***% of Systems***	***N = 1,501***	***% of Systems***	***N=1,400***	***% of Systems***	***N = 1,369***	***% of Systems***
MHI < 60% state's MHI	538	18.5	203	13.5	335	23.9	304	22.2
MHI > 60% state MHI but < 80% state's MHI	625	21.5	306	20.4	319	22.8	319	23.3
MHI > 80% state's MHI	1,719	59.3	992	66.1	727	51.9	727	53.1
Unknown	19	0.7	0	0	19	1.4	0	1.4
	N = 2,901	***% of Systems***	***N = 1,501***	***% of Systems***	***N=1,400***	***% of Systems***	***N = 1,369***	***% of Systems***
Publicly owned System	1,070	36.9	713	47.5	357	25.5	341	24.9
Privately owned System	1,831	63.1	788	52.5	1,043	74.5	1,028	75.1
	N = 2,901	***% of Systems***	***N = 1,501***	***% of Systems***	***N=1,400***	***% of Systems***	***N = 1,369***	***% of Systems***
Northern California	479	16.5	219	14.6	260	18.5	255	18.6
Bay Area	362	12.5	198	13.2	164	11.7	163	11.9
Eastern Sierras	199	3.6	96	6.4	103	7.3	100	7.3
Northern Sierras	170	5.9	74	4.9	96	6.6	92	6.7
San Joaquin Valley	654	22.5	282	18.8	372	26.6	361	26.4
Central Coast	374	12.9	203	13.5	171	12.2	167	12.2
Los Angeles/So.Cal	389	13.4	275	18.3	114	8.1	113	8.3
Inland Empire	274	9.4	154	10.3	120	8.6	118	8.6
	**Mean ± SD**		**Mean ± SD**		**Mean ± SD**		**Mean ± SD**	
	**N = 2,901**		**N = 1,501**		**N = 1,400**		**N = 1,369**	
% People of Color	40.7 ± 26.5		42.4 ± 26.5		38.9 ± 26.4		39.2 ± 26.4	
% Renters	33.8 ± 18.0		34 ± 16.4		33.7 ± 19.7		33.9 ± 19.6	
% Under 2X Poverty	35.9 ± 18.4		34.6 ± 17.7		37.2 ± 19.2		37.2 ± 19.2	

S5 Table B. Results of logarithmic predictions of missing data within size categories, by potential confounders of missing affordability data.

Each row by column cell reflects a unique binomial regression equation results. Significant results (alpha < 0.05) presented.

System Size (People Served)	N with Data / N no Data	Region *reference = Northern California*	Primary Source *reference = Surface water (SW)*	System Ownership *reference = Public*	% Renters	% Under 2x Poverty	% People of Color
**<500**	1,151 / 661	Nor Cal☆ OR=1.97***<br>Bay Area☆ OR=0.61**<br>C. Coast☆ OR=0.59***	GW☆ OR=1.44*	Public☆ OR=1.35**<br>Private☆ OR=1.37*	☆OR=1.01***	☆OR=1.01***	☆OR=1.01**
**501-3,300**	143 / 304	Nor Cal OR=0.43***	SW☆ OR=0.33***<br>GW☆ OR=1.63*	Public OR=0.43***	--	--	--
**3,301-10,000**	58 / 166	Nor Cal OR=0.15***<br>Nor Sierra OR=4.00*<br>SJV OR=3.61*	SW OR=0.29***	Public OR=0.41***<br>Private OR=0.43*	☆OR=1.02**	--	☆OR=1.01*
**10,000+**	48 / 370	Nor Cal OR=0.13**	☆GW OR=0.09***<br>☆SW OR=2.57**	☆Public OR=0.16***<br>☆Private OR=0.14**	☆OR=1.02*	--	--

* p < 0.05; ** p < 0.01; *** p < 0.001

OR = odds ratio, or, the odds of a system having missing data. OR > 1 and significant at alpha <0.05 means a significant increase in odds of a system having missing data. OR <1 and significant at alpha < 0.05 means a significant decrease in the odds of a system having missing data. ☆ = Mann Whitney's U test (for continuous variables) or Chi-squared ($\chi 2$) test (for categorical variables) was significant at alpha <0.05. Significant Mann Whitney's U test indicates a location shift greater or less than zero between the rank sums of systems missing data and those not missing data. Significant $\chi 2$ test indicates a rejection of the null that systems with missing data are as likely to have the same frequency of systems in each region/ownership/primary source distribution as those systems without missing data. For Intermediate and Large systems, low counts of systems by region meant that $\chi 2$ tests were not stable and therefore we conducted Fisher's exact tests using simulated p-values. In neither system size category were the count distributions significantly different between systems with and without missing data.
