# Supplementary material for: Water affordability and human right to water implications in California
Source: PLoS One. 2021 Jan 20;16(1):e0245237. doi: 10.1371/journal.pone.0245237 (PMC7816992; doi:10.1371/journal.pone.0245237)
Supplement: S6 File — (PDF) [file pone.0245237.s006.pdf]

PLOS ONE

Water affordability and human right to water implications in California

Jessica J. Goddard[1,2], Isha Ray[1], Carolina L. Balazs[2]
[1] Energy & Resources Group, University of California, Berkeley, California, United States of America
[2] Office of Environmental Health Hazard Assessment, California Environmental Protection Agency, Oakland, California

S6 Text. F-Test for Affordability Ratio Models With and Without System Size

S6 Table shows the results of the omnibus F-test between systems with and without system size. As the focus of this study is on the analysis of affordability ratios by system size, we present estimates and p-values only for water system size category but indicate which variables we adjusted for in the final model. Because AR_{CP}, and AR_{DP} are perfectly correlated in each system–DP is half the CP–we present results for AR_{CP} only.

S6 Table. General multiple linear regression models for affordability ratios, with and without size categories (n=1,501).

Model log-transformed affordability ratios; coefficients are back-transformed in table.

	AR_{MHI}		AR_{CP}	
	**Model 1 (without size category)**	**Model 2 (with size category)**	**Model 1 (without size category)**	**Model 2 (with size category)**
(Intercept)		0.57 (0.06)***		2.08 (0.06)***
pop bin		*Reference*		*Reference*
25-500		*Reference*		*Reference*
501-3,300		0.92 (0.05)		0.89 (0.04)**
3,301-10,000		0.72 (0.06)***		0.73 (0.06)***
10,000+		0.62 (0.06)***		0.63 (0.05)***
Ownership	**x**	**x**	**x**	**x**
% Under 2x poverty	**x**	**x**	**x**	**x**
% Renters	**x**	**x**	**x**	**x**
% People of Color	**x**	**x**	**x**	**x**
Primary Water Source	**x**	**x**	**x**	**x**
Region	**x**	**x**	**x**	**x**
Observations	1501	1501	1501	1501
R^2 / R^2 adjusted	0.212 / 0.206	0.253 / 0.245	0.120 / 0.112	0.166 / 0.158
AIC	2906.867	2834.371	2765.325	2690.02
F-test	SS = 30.481	F = 26.575***	SS = 30.659	F = 26.732***

Standard error in parentheses, based on log-transformed affordability ratios

* $p < 0.05$; ** $p < 0.01$; *** $p < 0.001$
