# Supplementary material for: Water affordability and human right to water implications in California
Source: PLoS One. 2021 Jan 20;16(1):e0245237. doi: 10.1371/journal.pone.0245237 (PMC7816992; doi:10.1371/journal.pone.0245237)
Supplement: S7 File — (PDF) [file pone.0245237.s007.pdf]

PLOS ONE

Water affordability and human right to water implications in California

Jessica J. Goddard[1,2], Isha Ray[1], Carolina L. Balazs[2]
[1] Energy & Resources Group, University of California, Berkeley, California, United States of America
[2] Office of Environmental Health Hazard Assessment, California Environmental Protection Agency, Oakland, California

S7 Text. Sensitivity Analysis Results

S7 Tables A and B show crude means, standard deviations, adjusted means, and 95% confidence intervals for affordability ratios at county poverty level and deep poverty level, respectively. As the overall result trends for affordability ratios for households earning median income levels did not change across the sensitivity analyses, these results are not shown. The tables demonstrate results of adjusted mean estimates and post-hoc tests for the complete case sample (n = 1,501), the sample less water systems identified as potentially unreliable census data (n=1,447, described in S4), and the sample less water systems identified in the potential water bill outliers assessment (n=1,408, described in S2). Post-hoc tests were conducted after generalized linear models showed a significant difference across affordability ratios for the main effect of interest (water system size), as indicated by F-tests shown in S6 Table.

As is clear in both tables, removing water systems with unreliable census data and potential water bill outliers results in more differentiation among mean affordability ratios across water system size categories. However, the overall trend–that ratios increase (i.e. become more unaffordable) as system size decreases–holds true across the complete sample and the sensitivity analyses.

S7 Table A. Sensitivity analysis for affordability ratios for households earning county poverty level (AR_{CP})‡.

	Affordability Ratio for Households at or Below County Poverty Level HHC_P (%)					
	Complete case (n=1,501)		Complete case less unreliable census (n=1,447)		Complete case less potential outliers (n=1,408)	
**Water System Size (pop.)**	**Crude means (SD)**	**Adjusted means (95% CI)**	**Crude means (SD)**	**Adjusted means (95% CI)**	**Crude means (SD)**	**Adjusted means (95% CI)**
**25-500**	2.8 ± 2.2[a]	2.2 (2.0, 2.3)	2.8 ± 2.2[a]	2.2 (2.1, 2.3)	2.6 ± 1.4[a]	2.2 (2.1, 2.3)
**501-3,300**	2.3 ± 1.6[a]	1.9 (1.8, 2.1)	2.2 ± 1.6[b]	1.9 (1.8, 2.0)	2.2 ± 1.3[b]	1.9 (1.8, 2.1)
**3,301-10,000**	1.7 ± 0.9[b]	1.6 (1.4, 1.7)	1.7 ± 0.7[c]	1.6 (1.4, 1.7)	1.8 ± 0.8[c]	1.7 (1.5, 1.8)
**10,000+**	1.5 ± 0.7[b]	1.3 (1.3, 1.5)	1.5 ± 0.7[c]	1.4 (1.3, 1.5)	1.5 ± 0.7[d]	1.5 (1.4, 1.5)

‡ Results are rounded to the tenth of a decimal for percentages. For adjusted means, all data were log transformed for statistical tests and back-transformed for the table. For each measure shown, means that share the same letter column-wise are not significantly different from one another based on Tukey's Honest Difference post-hoc tests on the generalized linear model of affordability ratios including measured confounders. Post-hoc letters were calculated using *multcomp* package in R (Version 3.5.1; R Development Core Team) and ordered to start comparisons with the highest mean value.

S7 Table B. Sensitivity analysis for affordability ratios for households earning county deep poverty level (AR_{DP})‡.

Affordability Ratio for Households at or Below Deep Poverty Level – HH_{DP} (%)						
	Complete case (n=1,501)		Complete case less unreliable census (n=1,447)		Complete case less potential outliers (n=1,408)	
Water System Size (pop.)	Crude means (SD)	Adjusted means (95% CI)	Crude means (SD)	Adjusted means (95% CI)	Crude means (SD)	Adjusted means (95% CI)
**25-500**	5.6 ± 4.3[a]	4.3 (4.1, 4.5)	5.6 ± 4.4[a]	4.3 (4.1, 4.5)	5.2 ± 2.8[a]	4.4 (4.2, 4.6)
**501-3,300**	4.5 ± 3.2[a]	3.8 (3.6, 4.1)	4.5 ± 3.2[b]	3.8 (3.6, 4.1)	4.4 ± 2.5[b]	3.9 (3.7, 4.1)
**3,301-10,000**	3.4 ± 1.7[b]	3.1 (2.8, 3.4)	3.4 ± 1.6[c]	3.1 (2.8, 3.4)	3.5 ± 1.7[c]	3.3 (3.1, 3.6)
**10,000+**	2.9 ± 1.4[b]	2.7 (2.5, 2.9)	2.9 ± 1.4[c]	2.7 (2.5, 2.9)	3.1 ± 1.3[d]	2.9 (2.7, 3.1)

‡ Results are rounded to the tenth of a decimal for percentages. For adjusted means, all data were log transformed for statistical tests and back-transformed for the table. For each measure shown, means that share the same letter column-wise are not significantly different from one another based on Tukey's Honest Difference post-hoc tests on the generalized linear model of affordability ratios including measured confounders. Post-hoc letters were calculated using *multcomp* package in R (Version 3.5.1; R Development Core Team) and ordered to start comparisons with the highest mean value.

S7 Table C shows results of sensitivity analysis for households earning at or below county poverty levels across the sample (n=1,501), the same less unreliable income data (n=1,274), the full water system list with water system boundaries to estimate poverty levels (n=2,882), and the full water system list with water system boundaries less unreliable income data (n=2663). Removing unreliable data reduces differences across system size categories in household poverty indices for systems in the sample, but not for systems in the full water system list. Trends did not change for households earning at or below deep poverty levels and thus the results are not shown.

S7 Table C. Sensitivity analysis for household poverty index (HH_{CP}) ‡

	Households at or below County poverty level HH_{CP} (%)			
**System Size** (People in System)	**Affordability Sample (n=1501)**	**Affordability sample less unreliable (n=1,274)**	**Water Systems with Boundaries (n=2882)**	**Water Systems with Boundaries less unreliable (n=2,663)**
**Very small** (<500)	22 ± 13[c]	21 ± 12[b]	24 ± 14[b]	24 ± 13[b]
**Small** (501-3,300)	28 ± 14[a]	27 ± 14[a]	28 ± 14[a]	27 ± 14[a]
**Medium** (3,301-10,000)	25 ± 11[a,b]	26 ± 11[a]	25 ± 11[a,b]	25 ± 11[a,b]
**Large** (10,001+)	24 ± 8[b]	24 ± 8[a]	24 ± 9[b]	24 ± 9[b]

‡ Results are rounded to the nearest integer. All data were square-root transformed for Welch's One-Way ANOVA and post-hoc statistical comparison tests and back-transformed for the table. For each measure shown, means that share the same letter column-wise are not significantly different from one another based on Games-Howell post-hoc tests for unequal variances. Post-hoc letters were calculated using userfriendlyscience and multcomp packages in R (Version 3.5.1; R Development Core Team) and ordered to start comparisons with the highest mean value.
