# Supplementary material for: Water affordability and human right to water implications in California
Source: PLoS One. 2021 Jan 20;16(1):e0245237. doi: 10.1371/journal.pone.0245237 (PMC7816992; doi:10.1371/journal.pone.0245237)
Supplement: S8 File — (PDF) [file pone.0245237.s008.pdf]

PLOS ONE

Water affordability and human right to water implications in California

Jessica J. Goddard[1,2], Isha Ray[1], Carolina L. Balazs[2]
[1] Energy & Resources Group, University of California, Berkeley, California, United States of America
[2] Office of Environmental Health Hazard Assessment, California Environmental Protection Agency, Oakland, California

S8 Table. Affordability Ratios by Common Affordability Thresholds

S8 Table summarizes common affordability thresholds and references, with results from study compared to these thresholds where applicable.

S8 Table. Common affordability thresholds and results for AR_{MHI}, AR_{CP}, AR_{DP}, where potentially comparable.

Affordability Ratio Threshold	Water Cost Included	Reference	% of Systems in Study Exceeding Threshold
**1.5% of MHI (specifically in disadvantaged communities)**	Drinking water services	[1,2]	AR_{MHI}: 18.7% (n = 281)<br>AR_{MHI}: 11.4% (n = 172 disadvantaged systems)
**2% of MHI**	Wastewater services	[3]	N/A
**2% of MHI**	Drinking water services	[4–6]	AR_{MHI}: 11.2% (n = 168)
**2.5% of MHI**	Drinking water services – for compliance purposes	[7,8]	AR_{MHI}: 6.3% (n = 95)
**3% of income (often referenced for drinking water alone and/ or for disposable incomes)**	Drinking water & wastewater services	[9–12]	AR_{CP}: 19% (n = 285)<br>AR_{DP}: 62% (n = 937)
**4.5% of MHI**	Drinking water & wastewater services	[13]	N/A
**5% of MHI**	Drinking water & wastewater services	[14,15]	N/A
**5% of discretionary income (for 20th income percentile)**	Drinking water services	[16–18]	AR_{CP}: 5.7% (n = 85)<br>AR_{DP}: 28.7% (n = 431)

S8 Table References

1. State Water Resources Control Board. Drinking Water State Revolving Fund Intended Use Plan State Fiscal Year 2018-2019. 2018. Available: https://www.waterboards.ca.gov/drinking_water/services/funding/documents/srf/iup_2018/dwsrf_iup_sfy2018_19_final.pdf
2. Pierce G, McCann H. Los Angeles County Community Water Systems: Atlas and Policy Guide. 2015.
3. US EPA. Combined Sewer Overflows - Guidance for Financial Capability Assessment and Schedule Development. 1997. Available: https://www3.epa.gov/npdes/pubs/csofc.pdf
4. Hanak E, Gray B, Lund J, Mitchell D, Chappelle C, Fahlund A, et al. Paying for Water in California. Public Policy Inst Calif. 2014.
5. Fong. AB 2334. 2012 pp. 1–3.

6. Christian-Smith J, Balazs CL, Heberger M, Longley K. Assessing Water Affordability: A Pilot Study in Two Regions of California. 2013.
7. EPA Science Advisory Board. Affordability Criteria for Small Drinking Water Systems: An EPA Science Advisory Board Report. 2002. Report No.: EPA-SAB-EEAC-03-004.
8. US EPA. Information for States on Developing Affordability Criteria for Drinking Water. 1998. Available: https://nepis.epa.gov/Exe/ZyPURL.cgi?Dockey=2000272B.TXT
9. UNOHCHR. The Right to Water 35, Fact Sheet No. 35. 2010. doi:ISSN 1014-5567
10. Barraqué B, Montginoul M. How to Integrate Social Objectives into Water Pricing. Global Issues in Water Policy. Springer; 2015. pp. 359–371. doi:10.1007/978-3-319-16465-6_18
11. Fitch M, Price H. Water Poverty in England and Wales. 2002. Available: http://www.cieh.org/uploadedFiles/Core/Policy/Environmental_protection/Water/waterpoverty.pdf
12. United Nations Development Program. Beyond Scarcity: Power, Poverty and the Global Water Crisis. Human Development Report. New York; 2006.
13. Mack EA, Wrase S. A Burgeoning Crisis? A Nationwide Assessment of the Geography of Water Affordability in the United States. PLoS One. 2017;12: 19. doi:10.1371/journal.pone.0169488
14. Banerjee SG, Morella E. Africa's water and sanitation infrastructure: access, affordability and alternatives. World Bank; 2011 Mar. doi:10.1596/978-0-8213-8457-2
15. Villumsen M, Jensen MH. The AAAQ Framework and the Right to Water: International indicators for availability, accessibility, acceptability and quality. 2014. Available: https://www.humanrights.dk/sites/humanrights.dk/files/media/dokumenter/udgivelser/aaaq/aaaq_contextualising_indicators_2014.pdf
16. Feinstein L. Measuring Progress Toward Universal Access to Water and Sanitation in California Defining Goals, Indicators, and Performance Measures. 2018. Available: http://pacinst.org/wp-content/uploads/2018/08/Measuring-Progress_Pacific-Institute_Sep-2018.pdf
17. Teodoro MP. Measuring Household Affordability for Water and Sewer Utilities. Am Water Work Assoc. 2018;110: 1–27. Available: http://mannyteodoro.com/wp-content/uploads/2014/03/MTeodoro_Affordability-Method-Working-Paper-Jul2017a.pdf
18. Teodoro MP. Water and sewer affordability in the United States. AWWA Water Sci. 2019;1: e1129. doi:10.1002/aws2.1129
